# Supplementary material for: Elucidating relationships between P.falciparum prevalence and measures of genetic diversity with a combined genetic-epidemiological model of malaria
Source: PLoS Comput Biol. 2021 Aug 19;17(8):e1009287. doi: 10.1371/journal.pcbi.1009287 (PMC8407561; doi:10.1371/journal.pcbi.1009287)
Supplement: S1 Appendix — (PDF) [file pcbi.1009287.s001.pdf]

Additional details on the model structure of `forward-dream`

Jason A. Hendry

May 2020

1 Implementation

In `forward-dream`, time is continuous and the occurrence of events is dictated by a competing Poisson process. The rate at which any event occurs, r_{total}, is equal to:

$$r_{total} = bN_v + \gamma h_1 + \epsilon v_1$$

which is the sum of the total biting rate across all vectors, the clearance rate across all infected hosts and the clearance rate across all infected vectors. The time until the next event is drawn from an exponential distribution with an expectation of $1/r_{total}$. Conditional on an event occurring, the type of event is sampled proportional to its fractional rate. For example, the probability of a given event being a biting event is:

$$P(bite|event) = \frac{bN_v}{r_{total}}$$

If a biting event occurs, the host and vector involved are sampled at random from the population, and the nature of parasite transmission depends on their infection status. Similarly, for clearance events, the host or vector to be cleared of infection (i.e. returned to the susceptible state) is chosen at random.

2 Parameterisation

`forward-dream` is completely specified by 17 parameter values (see Table 1 of the main text). Below we discuss how these parameters were set for the simulations presented in this study.

Biting rate (b)

The biting rate (b) gives the daily rate at which a single vector bites a host, such that $1/b$ gives the average time between bites. It is typically stated that $1/b$ ranges from 2-4 days, and common in modelling papers is to give an assumed value of $1/b = 3$. This is supported by some laboratory studies; for example [1] observed an approximately 3 day feeding and oviposition behaviour of *Anopheles gambiae* when offered daily blood meals and kept alone.

In `forward-dream` we set the default biting rate $b = 0.25$ which implies an average of 4 days between bites. We elected for a slightly lower default biting rate to partially compensate for the absence of an incubation time within the vector; bringing the average number of infectious bites delivered by a vector closer to what it would be were an incubation time included.

Vector-to-host (π_h) and host-to-vector (π_v) transmission efficiencies

The vector-to-host transmission efficiency (π_h) is the probability that a host becomes infected after being bitten by an infectious vector. An assembly and reanalysis of multiple sources of information on π_h has been conducted by [2]. The most direct source of information on transmission efficiency they identify comes from the control arm of drug or vaccine trials, where immunologically naive individuals are challenged with mosquitoes carrying *P. falciparum* [2]. Using data of this kind, [2] estimate a vector-to-host transmission efficiency of 55%. A second source of information comes from simultaneously collecting data on the force of

infection (FOI, the rate at which hosts become infected per unit time) and the entomological inoculation rate (EIR, the average number of infectious bites received per host per unit time). The ratio $\frac{FOI}{EIR}$ is then taken to equal the vector-to-host transmission efficiency [2]. A study of this type conducted in Saradidi, Kenya, estimated the vector-to-host transmission efficiency in children to be 7.5% [3]. Reanalysis by [2], fitting three different mathematical models (unrooted linear, linear, and a model accommodating for heterogeneous biting) to observations of $\frac{FOI}{EIR}$ collected through time, produced estimates of 5%, 2% and 27% (for the three models, respectively). A key conclusion of [2] was that transmission efficiency declines at higher transmission intensities (in particular when $EIR > 10$), and that this trend can be fit well using a heterogeneous biting model with $\pi_h = 0.55$. However, when models without heterogeneous biting are used, $\pi_h = 0.55$ produces FOI values that are significantly above observations; lower values of transmission efficiency provide a better fit to the data.

As `forward-dream` does not model heterogeneous biting, we set the default vector-to-host transmission efficiency to 10% ($\pi_h = 0.1$), roughly in the middle of the raw estimates reported by [2].

The host-to-vector transmission efficiency (π_v) is the probability that a vector becomes infected after biting an infectious host. Analysing 23-years of longitudinal data collected on the prevalence of infection in both hosts and vectors in Dielmo, Senegal, [4] produced estimates of π_v. Similarly to the vector-to-host transmission efficiency, [4] found that the π_v was lower in periods of higher transmission: ranging from ~ 0.18 at a parasite prevalence (PR) of 0.2, to ~ 0.03 at $PR = 0.7$ (for *Anopheles gambiae s.l.*).

In `forward-dream` we set the default host-to-vector transmission efficiency to 10% ($\pi_h = 0.1$).

Host infection clearance rate (γ)

The host infection clearance rate, γ, is the daily rate at which hosts in the infected state (H_1) return to the susceptible state (H_0). The host infection clearance rate is modelled as a constant, and so the duration of host infection is exponentially distributed with a mean of $1/\gamma$. Recall that in `forward-dream`, both singly- and multiply infected hosts are in the H_1 state. As γ is the same for all hosts in the H_1 state, multiple infection has no effect on the duration of host infection in `forward-dream`; all hosts have the same daily rate of clearance γ, regardless of the composition of their infection.

Estimate (days)	Study Type	Notes	Reference
211.6	Malariatherapy		[5]
54.9	*msp2* analysis	Gamma fit	[6]
139.9	*msp2* analysis	Weibull fit	[6]
205.3	*msp2* analysis	lognormal fit	[6]
219.7	*msp2* analysis	Exponential fit	[6]
152	*msp2* analysis		[7]
155	*msp2* analysis	<1 yo.	[8]
256	*msp2* analysis	1-2 yo.	[8]
257	*msp2* analysis	3-4 yo.	[8]
319	*msp2* analysis	5-9 yo.	[8]
176	*msp2* analysis	10-19 yo.	[8]
129	*msp2* analysis	20-39 yo.	[8]
126	*msp2* analysis	40-59 yo.	[8]
131	*msp2* analysis	>59 yo.	[8]

Table 1: A selection of estimates of the duration of *P. falciparum* infection. Estimates from [6; 7; 8] are all from the study of *msp2* alleles in Northern Ghana, but analyzed using different methods. In [8] estimates were aged-stratified. Horizontal lines separate estimates from individual studies. Abbreviations: yo., years old.

Studies examining the duration of untreated *P. falciparum* infections are scarce on account of the medical obligation to treat [5]. In the mid-1900s in the USA, the recommended treatment for individuals with

neurosyphilis was deliberate infection with *P. falciparum* malaria, and analysis of the progression of these infections has become an important source of information about untreated infection duration. Seeking to understand both the average duration of untreated infection and the best fitting distributional form, [5] analysed data from 54 such malariatherapy patients who were inoculated with *P. falciparum* either by sporozoites or infected blood, and were subsequently followed by daily microscopic examination. They observed a mean infection duration of 211.6 days [5]. It is also notable that they found that both the Gompetz and Weibull distributions provided better fits than the exponential model [5]. This data source has significant caveats, however, including that the adults involved were all naïve and co-infected with syphilisis, and the strains used were selected for having a low clinical virulence and the greatest curative properties [9]. Ultimately, the extent to which these infections reflect those developed in natural settings is unclear.

A second source of data on untreated infections comes from a study conducted in the Kassena-Nankana District of Northern Ghana in 2000-2001, a region with very high transmission ($EIR > 300$) [10]. In this study, approximately 300 asymptomatic individuals positive for malaria were tracked for a ten month period, where every two months blood samples were taken and both microscopic and PCR analysis of the *msp2* gene was performed [10]. The analysis of the *msp2* gene allowed for the tracking of individual parasite genotypes in a context where most individuals were multiply infected. A variety of authors have analysed this data source using different methods, and estimates of the duration of infection range from as low as 55 to as high as 319 days (see Table 1).

The review by [11] highlights other sources of information on infection duration, including accidental cases of malaria infection by blood transfusion, and provides evidence that in some cases a *P. falciparum* can last for multiple years.

For `forward-dream`, we set the default host infection clearance rate $\gamma = 0.005$, corresponding to an average duration of infection of 200 days. This is Macdonald's original estimate of infection duration [12], and broadly consistent with the estimates described above.

Vector infection clearance rate (ϵ)

In `forward-dream`, the vector infection clearance rate, ϵ, is the daily rate at which vectors in the infected state (v_1) return to the susceptible state (v_0). In the Ross-Macdonald model, it is classically assumed that vectors do not clear infections, but rather that the flow from v_1 to v_0 is driven by the death of infected vectors and their (instantaneous) replacement by susceptible vectors (reviewed in [13]). This allows for the vector population size (N_v) to be kept fixed. It further implies that ϵ represents the daily probability of death for infected vectors. As ϵ is a constant, the duration of vector lifespans is exponentially distributed with a mean of $1/\epsilon$. Consequently, vectors do not have an increased probability of death with age (they do not experience senescence); the common justification for this is that mosquitoes die too rapidly of other causes to die as a consequence of aging.

To set ϵ, we looked for data on either lifespan or the daily survival rate (commonly denoted p) of *Anopheles* mosquitoes from the field. Since we are looking to parameterise infected mosquitoes, we are particularly interested in data on female *Anopheles* mosquitoes. We note that there are a large variety of factors that influence mosquito survival rate in nature – including the climate/weather, most pertinently the temperature and humidity, the species and activities of the mosquito, and the presence of parasites and predators – and that these factors likely vary substantially through time and across different geographies [14]. Thus, in malaria endemic areas there is likely variation in ϵ through space in time.

A relatively straightforward approach to estimating the lifespan of *Anopheles* mosquitoes is by breeding them in controlled laboratory environments or outdoor cages. However, estimates of this kind are generally thought to be inflated, as many common sources of mortality that would act on wild vectors are not in effect [15]. [1] found that the maximum lifespan of a laboratory colony *A. gambiae* mosquitoes, fed on a 10% sucrose solution, was 16 days at 27°C and 34 days at 22°C ($p = 0.94$ and $p = 0.97$, respectively). Given that mortality in laboratory experiments is driven primarily by senescence, whereas in the Ross-Macdonald model it is implicitly assumed that mortality due to senescence is negligible, we consider these estimates unsuitable for parameterising `forward-dream`.

In general, the lifespan or survivorship of wild vectors cannot be measured directly [14]. However, a multitude of methods have been developed to provide indirect estimates. Below we briefly outline two general types of estimation approaches, and in Table 2 we provide a selection of estimates arising from them.

For a more detailed discussion and additional estimates, the reader should consult Chapter 13 of [16] which covers the subject extensively.

p	Method	Location	Species	Details	Ref.
0.74	Mark-release-recapture	Burkina Faso	*An. gambiae*		[17]
0.77	Age-composition	Tanzania	*An. gambiae*	Michenga	[18]
0.78	Age-composition	D.R.C.	*An. gambiae*	Urban	[19]
0.81	Age-composition	Senegal	*An. arabiensis*		[20]
0.82	Age-composition	Kenya	*An. gambiae*		[15]
0.83	Age-composition	Nigeria	*An. gambiae*		[21]
0.84	Age-composition	Tanzania	*An. gambiae*	Namawala	[18]
0.84	Mark-release-recapture	Tanzania	*An. gambiae*		[22]
0.87	Age-composition	Kenya	*An. funestus*		[15]
0.91	Age-composition	Tanzania	*An. gambiae*		[23]
0.91	Age-composition	D.R.C.	*An. gambiae*	Rural	[19]
0.93	Age-composition	Uganda	*An. gambiae*		[23]

Table 2: A selection of daily survival rate (p) estimates for *Anopheles* vectors. Note that in `forward-dream` the vector infection clearance rate is $\epsilon = 1 - p$.

The first type of approach is based on Mark-release-recapture experiments. Here, mosquitoes are marked, originally with paint or a radioisotope, and released in a natural environment [22]. It has been observed that the number of marked mosquitoes recaptured on every subsequent day declines, and it assumed this is due to mortality [22]. From this decline, daily mortality can be estimated in a variety of ways (reviewed in [16]). This type of approach can be challenged by low recapture numbers, changes in mosquito survival or behaviour on account of the marking, and the confounding of mortality with other reasons for mosquito loss, such as dispersal.

A second approach looks for indicators of the age-composition of the mosquito population, typically with respect to developmental stages. For example, whether a mosquito is parous (has laid eggs) or nulliparous (has not laid eggs) can be estimated by dissection [23]. If an assumption is made about the duration of time until egg laying, the daily survival rate can be estimated by the proportion parous [23]. A great diversity of methods of this type exists, focusing different age-composition indicators (reviewed in [16]). In general, they all involve making assumptions about (and are sensitive to) the duration of the developmental stage(s) under consideration.

Overall, estimates of the survival rate (p) of *Anopheles* mosquitoes typically fall between 0.75 and 0.95 (see Table 2). For `forward-dream`, the vector infection clearance rate is $\epsilon = 1 - p$ and we set the default value at 0.2, corresponding to a survival rate of 0.8.

Number of sub-compartments in hosts (n_h) and vectors (n_v)

An individual host can harbour up to 10^9-10^{11} *P. falciparum* parasites [24]. However, in all the simulations described here, n_h (and n_v) are set to twenty. This is motivated primarily by reducing computational costs, but the simplification can also be justified biologically. First, even if an individual host contains 10^9-10^{11} parasites, *P. falciparum* replicates clonally within the host and so the majority of these will have near-identical genomes. A host may, however, be infected by multiple distinct parasite strains, generating a mixed infection. Importantly, setting $n_h = 20$ still accommodates for this possibility. Second, parasite genomes that exist at a frequency much less than 1/20 of the population are unlikely to produce substantial signal in genetic data. For example, with whole-genome sequencing data, sequencing depths are typically $\leq$ 100X and so parasites comprising less than 1/20 of the infection will be represented by only a handful of reads.

Mutation and drift rates in the host (θ_h, d_h) and vector (θ_v, d_v)

We have combined the mutation and drift rate parameterisation into a single section because in `forward-dream` mutational events occur conditional on drift events, and thus the parameters are related. In particular, considering hosts, drift events occur at a fixed constant rate d_h such that for any time interval δt the number of drift events will be drawn from $\sim Poi(\delta t d_h)$. Note that `forward-dream` implements a Moran model, such that for each drift event two sub-compartments are selected at random, and one is duplicated while the other is deleted. With probability $\theta_h N_{snps}$, each of these events will be instead a mutational event. The same occurs for vectors with rates d_v and θ_v.

The malaria parasite genome in `forward-dream` is designed to represent a collection of SNPs, and there are at least two sources of information on the single-nucleotide variant (SNV) mutation rate of *P. falciparum*. The first is from clone-tree experiments, where *P. falciparum* strains are cultured *in vitro* and accumulated mutations are detected by whole-genome sequencing. From these experiments several estimates of the mutation rate of *P. falciparum* during the erythrocytic phase have been produced, typically on the order of $\sim 10^{-10}$ [25; 26]. A second approach is to look at sequence divergence from a related *Plasmodium* species, such as *Plasmodium riechenowi* [27]. Here, the estimated mutation rate corresponds to the entire generation of *P. falciparum*, i.e. including both the host and vector phases. We have tabulated a selection of these mutation rates below. We are unaware of any mutation rate estimates specific to the vector phase.

SNV Rate	Value	Strain	Method	Ref.
per gen. per bp	6.88 x 10^{-10}	Wild	Phylogenetic cf. to *P. riechenowi*	[27]
per gen. per bp	4.90 x 10^{-10}	Wild	Phylogenetic cf. to *P. riechenowi*	[27]
per ieg per bp	4.07 x 10^{-10}	3D7	Clone-tree	[25]
per ieg per bp	3.63 x 10^{-10}	Dd2	Clone-tree	[25]
per ieg per bp	3.78 x 10^{-10}	HB3	Clone-tree	[25]
per ieg per bp	2.10 x 10^{-10}	3D7	Clone-tree	[26]
per ieg per bp	3.20 x 10^{-10}	Dd2	Clone-tree	[26]
per ieg per bp	2.95 x 10^{-10}	HB3	Clone-tree	[26]
per ieg per bp	2.27 x 10^{-10}	KH-01	Clone-tree	[26]
per ieg per bp	1.64 x 10^{-10}	KH-02	Clone-tree	[26]
per ieg per bp	1.70 x 10^{-9}	3D7	Clone-tree	[28]
per ieg per bp	3.20 x 10^{-9}	Dd2	Clone-tree	[28]

Table 3: A selection of mutation rate estimates for *P. falciparum*. The two different estimates from [27] arise from differing assumptions about the speciation time between *P. falciparum* and *P. reichenowi* (5Mya or 7Mya.). Note that [28] estimates are an order of magnitude higher, but have been adjusted to include unobserved lethal mutations.

It was unclear to us how to use these mutation rates to parameterise `forward-dream`. Given that $n_h = 20$ and $n_v = 20$, each sub-compartment can be thought of as representing the consensus sequence of 1/20th of the parasite population, rather than a single genome. This implies that a mutational event in `forward-dream` is equivalent to an event whereby a novel variant reaches a frequency 1/20, within the host/vector. Note that this is not co-incident with the mutation occurring, but is related also to the rate of drift. In addition, though n_h and n_v are fixed in `forward-dream`, in reality the size of the parasite population fluctuates dramatically in both the vectors and the hosts over the course of an infection – going through exponential growth phases and bottlenecks in response to immune pressure.

For simplicity, we set the host and vector mutation and drift rates equally, to $d_h = 2$, $d_v = 2$ and $\theta_h = 0.0000125$, $\theta_v = 0.0000125$. Thus, for both hosts and vectors, a drift event occurs on average twice every day and, with $N_{snps} = 8000$, one in every ten drift events produces a mutation. Since there are ten sub-compartments in both the hosts and the vectors, this implies that an individual sub-compartment acquires a mutation once every one-hundred days. In the future, estimates of these parameters can be strengthened by the collection of longitudinal WGS data for *P. falciparum*.

Probability a given parasite genome passes through the host-to-vector (p_h) or vector-to-host bottleneck (p_v)

The two parameters p_h and p_v control the size of the bottleneck when parasites are being transmitted from the host to the vector, or vector to the host, respectively. In terms of the `forward-dream` model, p^v gives the probability that a given sub-compartment in the vector is selected to colonize a host sub-compartment (see main text). Biologically, we expect it to be determined by the processes that reduce the amount of genetic diversity found in the initial blood-stage infection, relative to that present in the salivary glands of the vector that initiated that infection. This would include the number of sporozoites that are transmitted to the host, how representative they are of the genetic diversity within the salivary glands, and what fraction of them succeed in invading hepatocytes.

By dissecting wild caught *Anopheles gambiae* salivary glands and quantifying the abundance of sporozoite-specific antigens (either with an ELISA or immunoradiometric assay), the average number of sporozoites within an infectious vector has been found to be around 4000 to 5000, although the distributions are highly variable with some mosquitoes found carrying tens of thousands of sporozoites [29; 30]. Estimates of the number of sporozoites delivered in a single bite are also variable. Using a membrane feeding assay, [31] found that a geometric mean of 4.9 sporozoites were delivered by *An. freeborni* and 11.3 sporozoites were delivered by *An. gambiae* mosquitoes. In rodent model systems, two studies found an average of 123 and 281 *Plasmodium berghii* sporozoites were injected in a single bite, though again the distributions were highly variable with over a thousand sporozoites delivered in some cases [32; 33]. Cumulatively these results suggest that, typically, only a small percentage of salivary gland sporozoites are delivered to the host [31]. Finally, analysing early blood stage infections, [34] deduced that roughly 20% of sporozoites injected into the host reach the liver stage and ultimately produce merozoites; further emphasising that only a very small percentage of all salivary gland sporozoites establish infection within the host.

However, we note that the numerical reduction in number of sporozoites may be significantly greater than the amount of genetic diversity that is lost, as this is critically dependent on the amount of genetic diversity in the original salivary gland population. Consider that if there are only four unique *P. falciparum* genomes within the salivary glands (which could occur if the vector harboured a single oocyst), then a minimum of only 4 sporozoites is needed to re-establish 100% of genetic diversity within the host. This would be possible even if only tens to hundreds of sporozoites were transmitted. Furthermore, we note that the rate of co-infection in *P. falciparum* has been found to be high: globally it is nearly equivalent to the rate of super-infection in mixed infections carrying two strains [35], and there is evidence that co-infection occurs regularly in high transmission settings [36], observations that would be unlikely if a substantial fraction of diversity was lost with transmission.

As a starting point, we set p_v to 0.2 and p_h to 0.2; though the biological processes impacting the value of p_h differ (it concerns the fraction of gametocytes that are imbibed by a vector), it is subject to similar uncertainties as p_v. As a consequence (and with n_h and n_v set to 20), a mean of four parasite genomes will pass through the bottleneck; though we note that in $\sim 40\%$ of cases it will be less than four. Studies analysing the genetic diversity present within the vector midgut and salivary glands could strengthen the estimates of these parameters.

Distribution of number of oocysts ($p_{oocysts}$)

As described in the main text, we model the number of oocysts that are generated during meiosis in an individual mosquito by a truncated geometric distribution:

$$N_{oocysts} \sim max[Geo(p_{oocysts}), 10]$$

$p_{oocysts}$ therefore represents the probability of having only a single oocyst, and we restrict the maximum number of oocysts to ten. The number of oocysts in wild-caught *Anopheles* mosquitoes can be determined by dissection. We used two references to set $p_{oocysts}$: (i) [29] caught *Anopheles gambiae s.l.* near Kisumu in Kenya, and Fig. 2 of that reference suggests an approximately geometric distribution with $p = 0.5$; (ii) [30] caught *Anopheles gambiae s.l.* near Basse in The Gambiae, and Fig. 4 of that reference appears roughly geometric, with a $p \leq 0.5$. Consequently, we set $p_{oocysts} = 0.5$ in `forward-dream`.

References

1. Fernandes L, Briegel H. Reproductive physiology of Anopheles gambiae and Anopheles atroparvus. J Vector Ecol. 2005;30(1):11–26.

2. Smith DL, Drakeley CJ, Chiyaka C, Hay SI. A quantitative analysis of transmission efficiency versus intensity for malaria. Nature Communications. 2010;1(1):108. doi:10.1038/ncomms1107.

3. Beier JC, Oster CN, Onyango FK, Bales JD, Sherwood JA, Perkins PV, et al. Plasmodium falciparum Incidence Relative to Entomologic Inoculation Rates at a Site Proposed for Testing Malaria Vaccines in Western Kenya. 1994;50(5):529–536. doi:https://doi.org/10.4269/ajtmh.1994.50.529.

4. Churcher TS, Trape JF, Cohuet A. Human-to-mosquito transmission efficiency increases as malaria is controlled. Nature communications. 2015;6:6054–6054. doi:10.1038/ncomms7054.

5. Sama W, Dietz K, Smith T. Distribution of survival times of deliberate Plasmodium falciparum infections in tertiary syphilis patients. Trans R Soc Trop Med Hyg. 2006;100(9):811–6. doi:10.1016/j.trstmh.2005.11.001.

6. Bretscher MT, Maire N, Chitnis N, Felger I, Owusu-Agyei S, Smith T. The distribution of Plasmodium falciparum infection durations. Epidemics. 2011;3(2):109–118. doi:https://doi.org/10.1016/j.epidem.2011.03.002.

7. Sama W, Owusu-Agyei S, Felger I, Vounatsou P, Smith T. An immigration-death model to estimate the duration of malaria infection when detectability of the parasite is imperfect. Stat Med. 2005;24(21):3269–88. doi:10.1002/sim.2189.

8. Felger I, Maire M, Bretscher MT, Falk N, Tiaden A, Sama W, et al. The Dynamics of Natural Plasmodium falciparum Infections. PLOS ONE. 2012;7(9):e45542. doi:10.1371/journal.pone.0045542.

9. McKenzie FE, Smith DL, O'Meara WP, Riley EM. Strain theory of malaria: the first 50 years. Adv Parasitol. 2008;66:1–46. doi:10.1016/s0065-308x(08)00201-7.

10. Owusu-Agyei S, Smith T, Beck HP, Amenga-Etego L, Felger I. Molecular epidemiology of Plasmodium falciparum infections among asymptomatic inhabitants of a holoendemic malarious area in northern Ghana. Trop Med Int Health. 2002;7(5):421–8. doi:10.1046/j.1365-3156.2002.00881.x.

11. Ashley EA, White NJ. The duration of Plasmodium falciparum infections. Malaria Journal. 2014;13(1):500. doi:10.1186/1475-2875-13-500.

12. Macdonald G. The analysis of malaria parasite rates in infants. Trop Dis Bull. 1950;47(10):915–38.

13. Smith DL, Battle KE, Hay SI, Barker CM, Scott TW, McKenzie FE. Ross, Macdonald, and a Theory for the Dynamics and Control of Mosquito-Transmitted Pathogens. PLOS Pathogens. 2012;8(4):e1002588. doi:10.1371/journal.ppat.1002588.

14. Bates M. The Natural History of Mosquitoes. Peter Smith; 1970.

15. Chege GM, Beier JC. Effect of Plasmodium falciparum on the survival of naturally infected afrotropical Anopheles (Diptera: Culicidae). J Med Entomol. 1990;27(4):454–8. doi:10.1093/jmedent/27.4.454.

16. Silver JB. Mosquito Ecology: Field Sampling Methods. Springer Netherlands; 2007. Available from: `https://books.google.co.uk/books?id=VM8MA4E_VT8C`.

17. Costantini C, Li SG, Della Torre A, Sagnon N, Coluzzi M, Taylor CE. Density, survival and dispersal of Anopheles gambiae complex mosquitoes in a west African Sudan savanna village. Med Vet Entomol. 1996;10(3):203–19. doi:10.1111/j.1365-2915.1996.tb00733.x.

18. Charlwood JD, Smith T, Billingsley PF, Takken W, Lyimo EOK, Meuwissen JHET. Survival and infection probabilities of anthropophagic anophelines from an area of high prevalence of Plasmodium falciparum in humans. Bulletin of Entomological Research. 1997;87(5):445–453. doi:10.1017/S0007485300041304.

19. Coene J. Malaria in urban and rural Kinshasa: the entomological input. Med Vet Entomol. 1993;7(2):127–37. doi:10.1111/j.1365-2915.1993.tb00665.x.

20. Vercruysse J. Estimation of the Survival Rate of Anopheles arabiensis in an Urban Area (Pikine–Senegal). Journal of Animal Ecology. 1985;54(2):343–350. doi:10.2307/4482.

21. type [; 1980]Available from: https://apps.who.int/iris/handle/10665/40316.

22. Gillies MT. Studies on the dispersion and survival of Anopheles gambiae Giles in East Africa, by means of marking and release experiments. Bulletin of Entomological Research. 1961;52(1):99–127. doi:10.1017/S0007485300055309.

23. Davidson G. Estimation of the survival rate of anopheline mosquitoes in nature. Nature. 1954;174(4434):792–3. doi:10.1038/174792a0.

24. White NJ. Antimalarial drug resistance. The Journal of clinical investigation. 2004;113(8):1084–1092. doi:10.1172/JCI21682.

25. Claessens A, Hamilton WL, Kekre M, Otto TD, Faizullabhoy A, Rayner JC, et al. Generation of antigenic diversity in Plasmodium falciparum by structured rearrangement of Var genes during mitosis. PLoS Genet. 2014;10(12):e1004812. doi:10.1371/journal.pgen.1004812.

26. Hamilton WL, Claessens A, Otto TD, Kekre M, Fairhurst RM, Rayner JC, et al. Extreme mutation bias and high AT content in Plasmodium falciparum. Nucleic Acids Res. 2017;45(4):1889–1901. doi:10.1093/nar/gkw1259.

27. Joy DA, Feng X, Mu J, Furuya T, Chotivanich K, Krettli AU, et al. Early origin and recent expansion of Plasmodium falciparum. Science. 2003;300(5617):318–21. doi:10.1126/science.1081449.

28. Bopp SER, Manary MJ, Bright AT, Johnston GL, Dharia NV, Luna FL, et al. Mitotic Evolution of Plasmodium falciparum Shows a Stable Core Genome but Recombination in Antigen Families. PLOS Genetics. 2013;9(2):e1003293. doi:10.1371/journal.pgen.1003293.

29. Beier JC, Onyango FK, Ramadhan M, Koros JK, Asiago CM, Wirtz RA, et al. Quantitation of malaria sporozoites in the salivary glands of wild Afrotropical Anopheles. Med Vet Entomol. 1991;5(1):63–70.

30. Collins FH, Zavala F, Graves PM, Cochrane AH, Gwadz RW, Akoh J, et al. First Field Trial of an Immunoradiometric Assay for the Detection of Malaria Sporozoites in Mosquitoes. 1984;33(4):538–543. doi:https://doi.org/10.4269/ajtmh.1984.33.538.

31. Beier JC, Beier MS, Vaughan JA, Pumpuni CB, Davis JR, Noden BH. Sporozoite transmission by Anopheles freeborni and Anopheles gambiae experimentally infected with Plasmodium falciparum. J Am Mosq Control Assoc. 1992;8(4):404–8.

32. Medica DL, Sinnis P. Quantitative Dynamics of ¡em¿Plasmodium yoelii¡/em¿ Sporozoite Transmission by Infected Anopheline Mosquitoes. 2005;73(7):4363–4369. doi:10.1128/IAI.73.7.4363-4369.2005

33. Jin Y, Kebaier C, Vanderberg J. Direct Microscopic Quantification of Dynamics of ¡em¿Plasmodium berghei¡/em¿ Sporozoite Transmission from Mosquitoes to Mice. 2007;75(11):5532–5539. doi:10.1128/IAI.00600-07

34. Bejon P, Andrews L, Andersen RF, Dunachie S, Webster D, Walther M, et al. Calculation of Liver-to-Blood Inocula, Parasite Growth Rates, and Preerythrocytic Vaccine Efficacy, from Serial Quantitative Polymerase Chain Reaction Studies of Volunteers Challenged with Malaria Sporozoites. The Journal of Infectious Diseases. 2005;191(4):619–626. doi:10.1086/427243.

35. Zhu SJ, Hendry JA, Almagro-Garcia J, Pearson RD, Amato R, Miles A, et al. The origins and relatedness structure of mixed infections vary with local prevalence of P. falciparum malaria. Elife. 2019;8. doi:10.7554/eLife.40845.

36. Nkhoma SC, Trevino SG, Gorena KM, Nair S, Khoswe S, Jett C, et al. Co-transmission of Related Malaria Parasite Lineages Shapes Within-Host Parasite Diversity. Cell Host Microbe. 2020;27(1):93–103.e4. doi:10.1016/j.chom.2019.12.001.
