# Supplementary figures and images for: Elucidating relationships between P.falciparum prevalence and measures of genetic diversity with a combined genetic-epidemiological model of malaria
Source: PLoS Comput Biol. 2021 Aug 19;17(8):e1009287. doi: 10.1371/journal.pcbi.1009287 (PMC8407561; doi:10.1371/journal.pcbi.1009287)

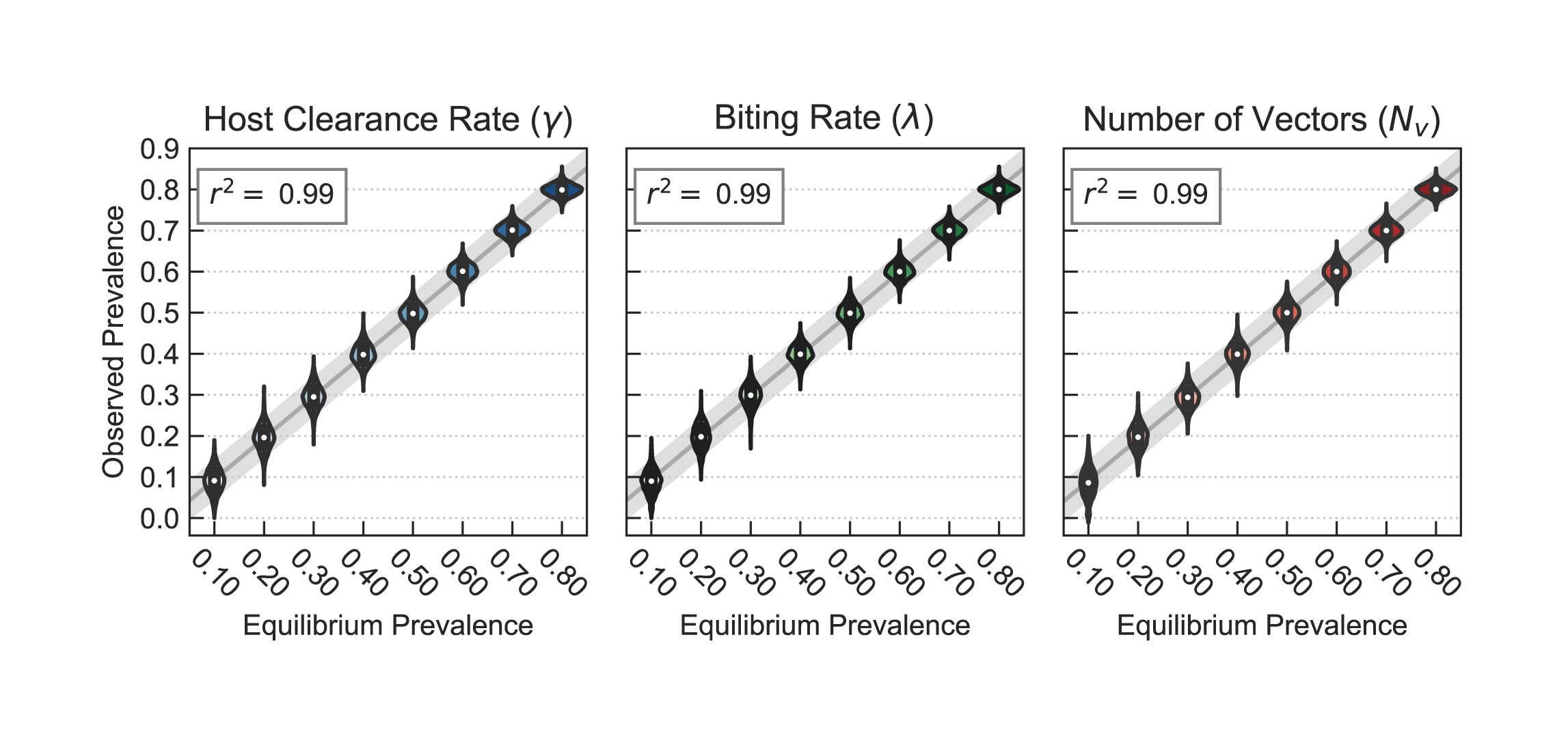

Supplement: S1 Fig — The epidemiological layer of forward-dream implements the Ross-Macondald model, where the host prevalence is a function of the rate parameters (see Eq 1). Violinplots summarize the prevalence values observed in forward-dream simulations with expected equilibrium prevalence values varying from 0.1 to 0.8 (computed using Eq 1) given on the x-axis. The different equilibrium prevalence values were achieved by varying either the host clearance rate (γ), the vector biting rate (b), or the number of vectors (Nv). The variance explained (r2) in an ordinary linear regression is shown at top-left of each plot. (TIF) [file pcbi.1009287.s002.tif]

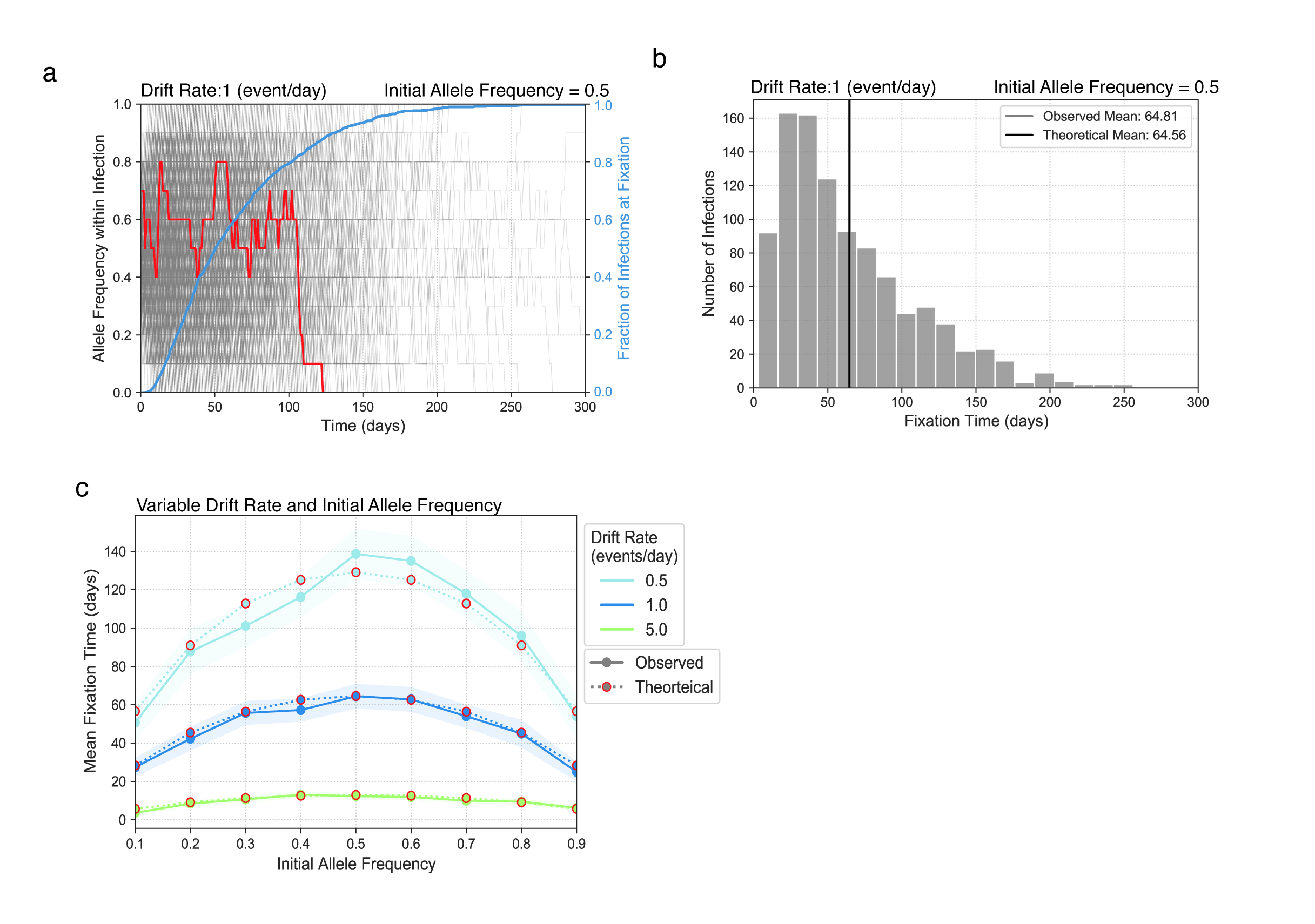

Supplement: S2 Fig — (a) The infection of a single host is evolved through time and the within-host alelle frequency of a given site is indicated by the red line. The site fixes around day 125. The experiment is repeated 1000 times (grey lines) and the fraction of infections fixed at a given time is indicated by the blue line. All experiments started with an initial allele frequency of 0.5 and a drift rate of 1/event per day. (b) Distribution of fixation times from (a). The observed mean (64.81 days) is very close to the theoretically expected mean from the Moran model (64.56 days). (c) The experiment in (a) is repeated but with different initial allele frequencies (x-axis) and three different drift rates (light blue, dark blue, and green line). In all cases, the observed mean fixation times are close to the theoretically expected times. Shading gives 95% confidence intervals for mean estimates. (TIF) [file pcbi.1009287.s003.tif]

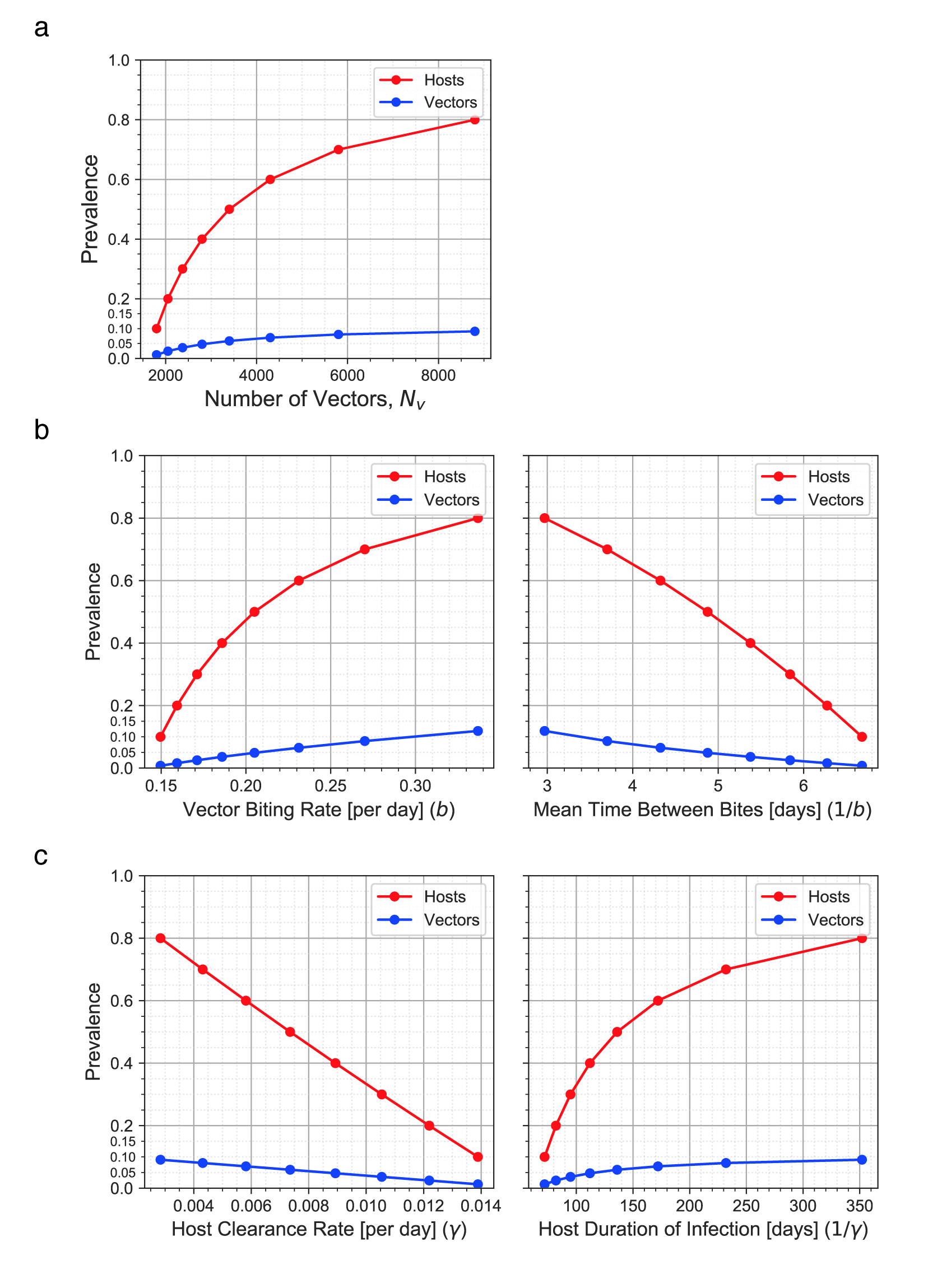

Supplement: S3 Fig — The parameter values of forward-dream are varied to produce simulations with equilibrium parasite prevalence values varying from 0.1 to 0.8. (a) Varying the number of vectors. Prevalence in hosts indicated in red, vectors in blue. Dots mark parasite prevalence values of 0.1 through 0.8. (b) Varying the vector biting rate b. Note 1/b gives the average time between successive bites, show in right plot. (c) Varying the host clearance rate (γ). Note 1/γ gives the average duration of host infection, shown in right plot. (TIF) [file pcbi.1009287.s004.tif]

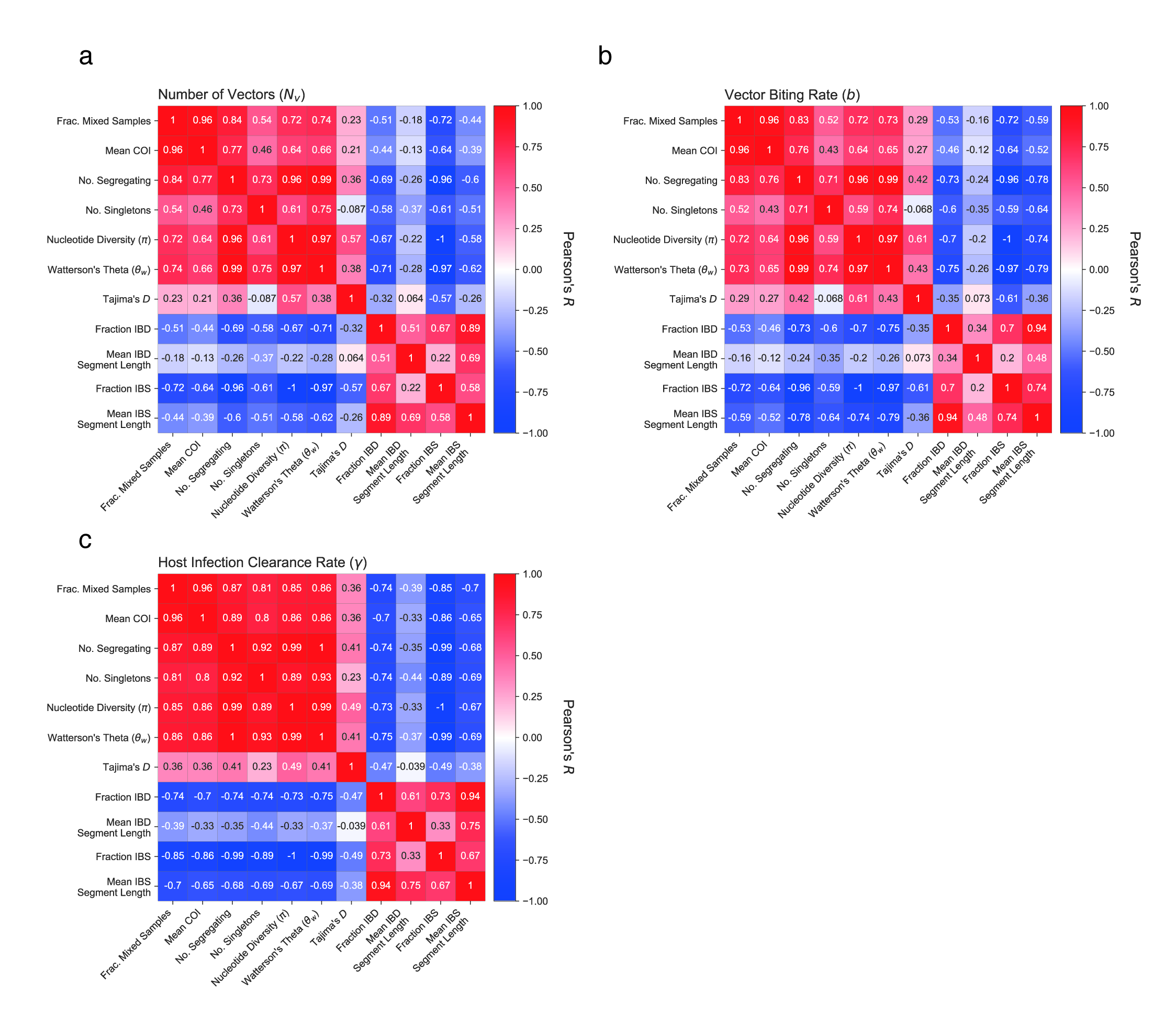

Supplement: S4 Fig — Matrices of Pearson’s Correlation Co-efficient (R) calculated between all pairs of genetic diversity statistics is shown. In panel (a) host prevalence was tuned to different values between 0.1 and 0.8 by varying the host clearance rate (γ); (b) by varying the vector biting rate (b); or (c) by varying the number of vectors Nv. In all cases there is significant co-linearity between different genetic diversity statistics. (TIF) [file pcbi.1009287.s005.tif]

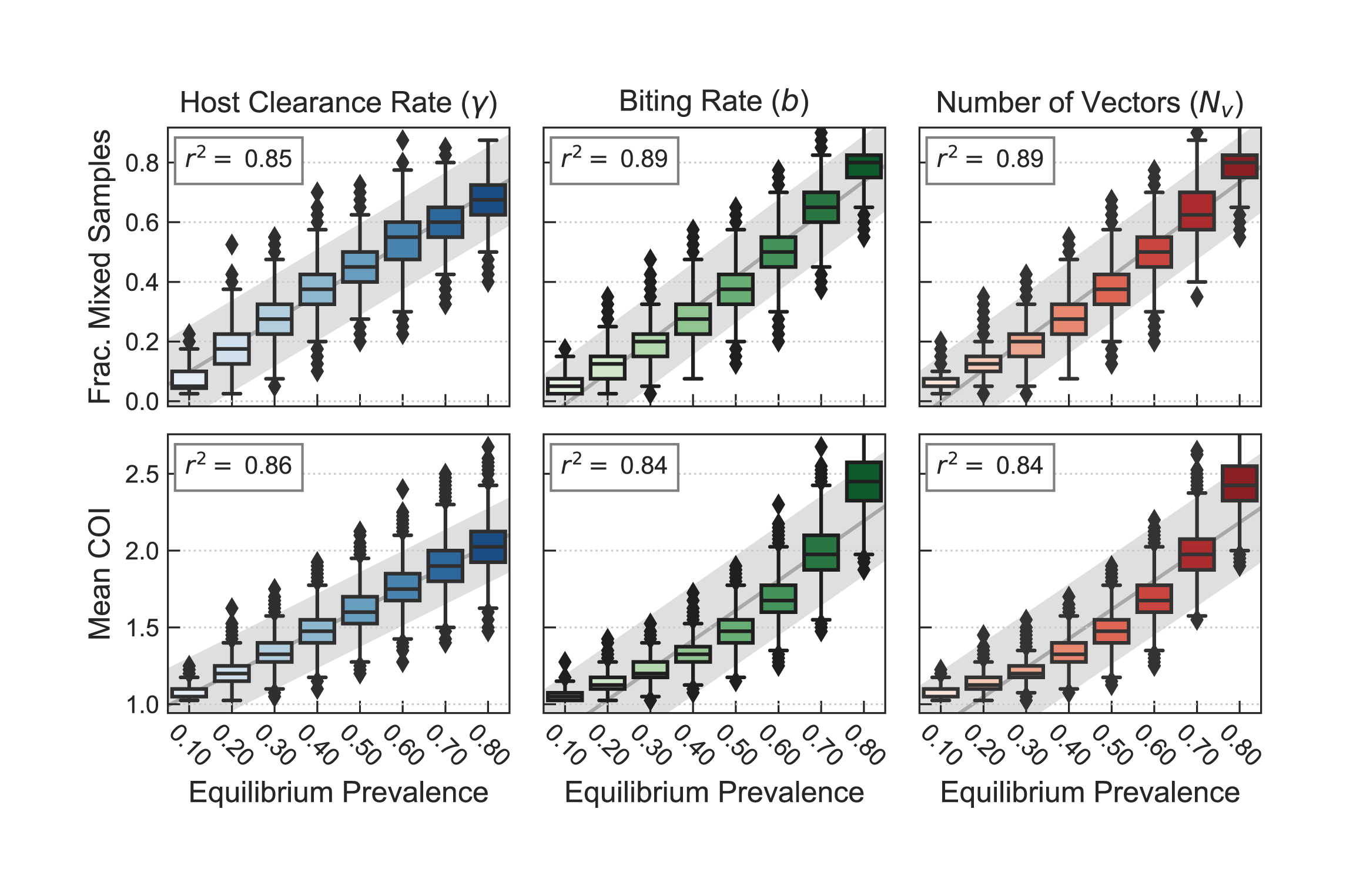

Supplement: S5 Fig — Distributions of mixed infection related genetic diversity statistics (y-axis), plotted for equilibrium parasite prevalence values tuned to between 0.2 and 0.8 (x-axis) in forward-dream simulations. Left, middle, and right columns show distributions when parasite prevalence is varied as a function of the host clearance rate (γ, in blue), vector biting rate (b, in green) or number of vectors (Nv, in green). Each boxplot contains the result of 30 replicate experiments, where the parasite genomes within 40 randomly selected hosts are collected at every 30 days for 10 years and are used to compute the genetic statistic of interest. The variance explained by ordinary least squares regression is given at top left, and line of best fit and confidence intervals indicated in grey. (TIF) [file pcbi.1009287.s006.tif]

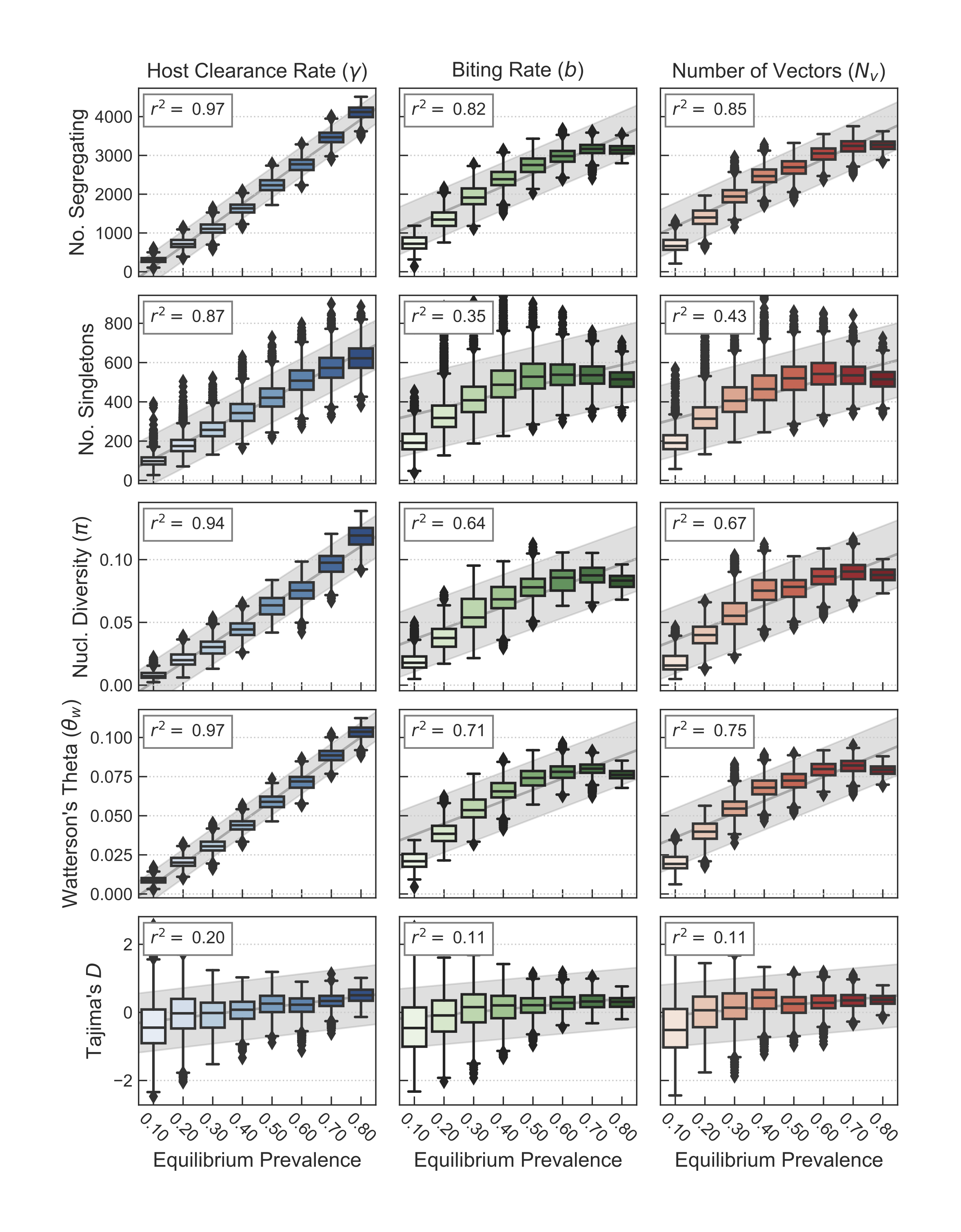

Supplement: S6 Fig — See S5 Fig for details. (TIF) [file pcbi.1009287.s007.tif]

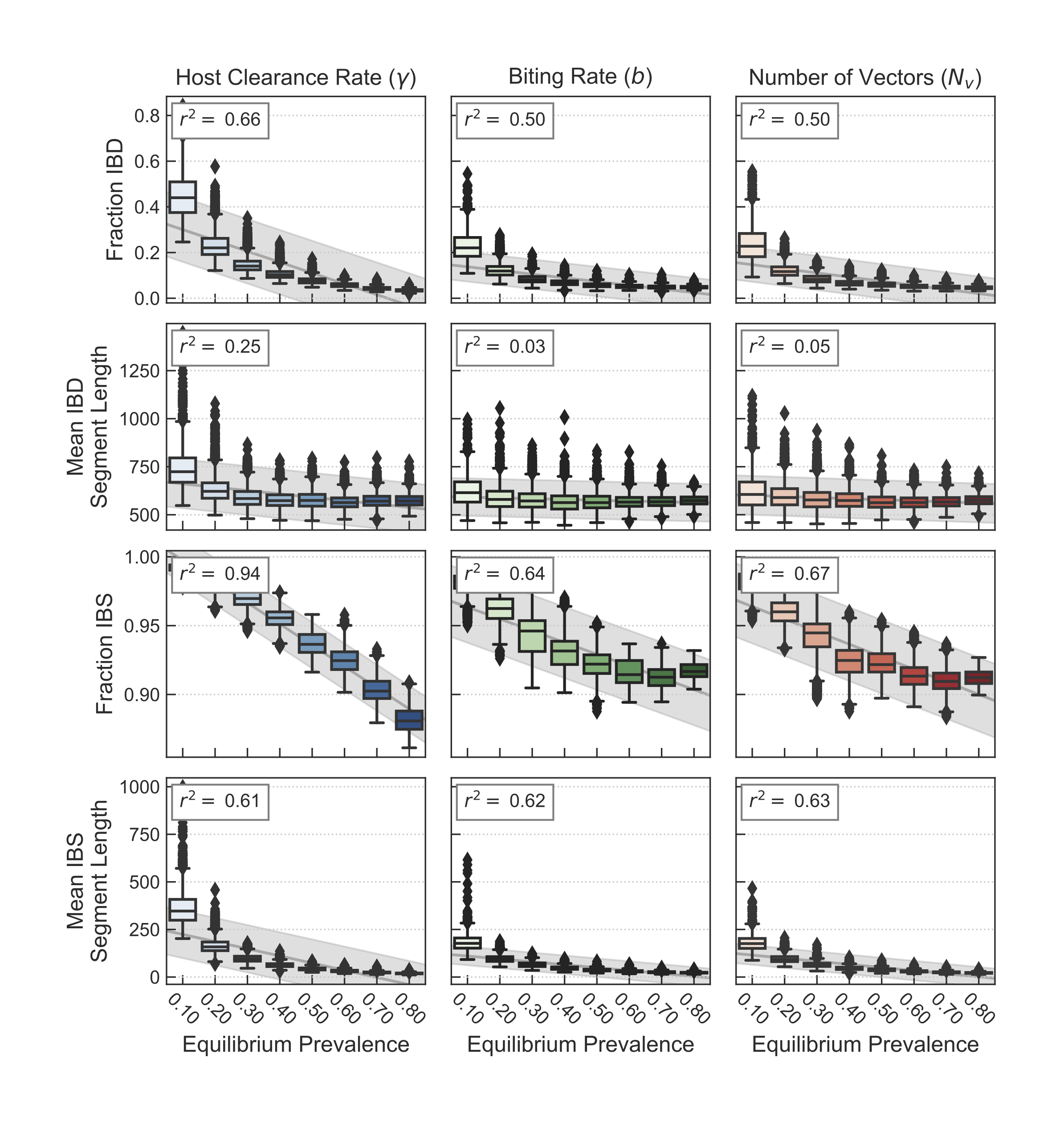

Supplement: S7 Fig — See S5 Fig for details. (TIF) [file pcbi.1009287.s008.tif]

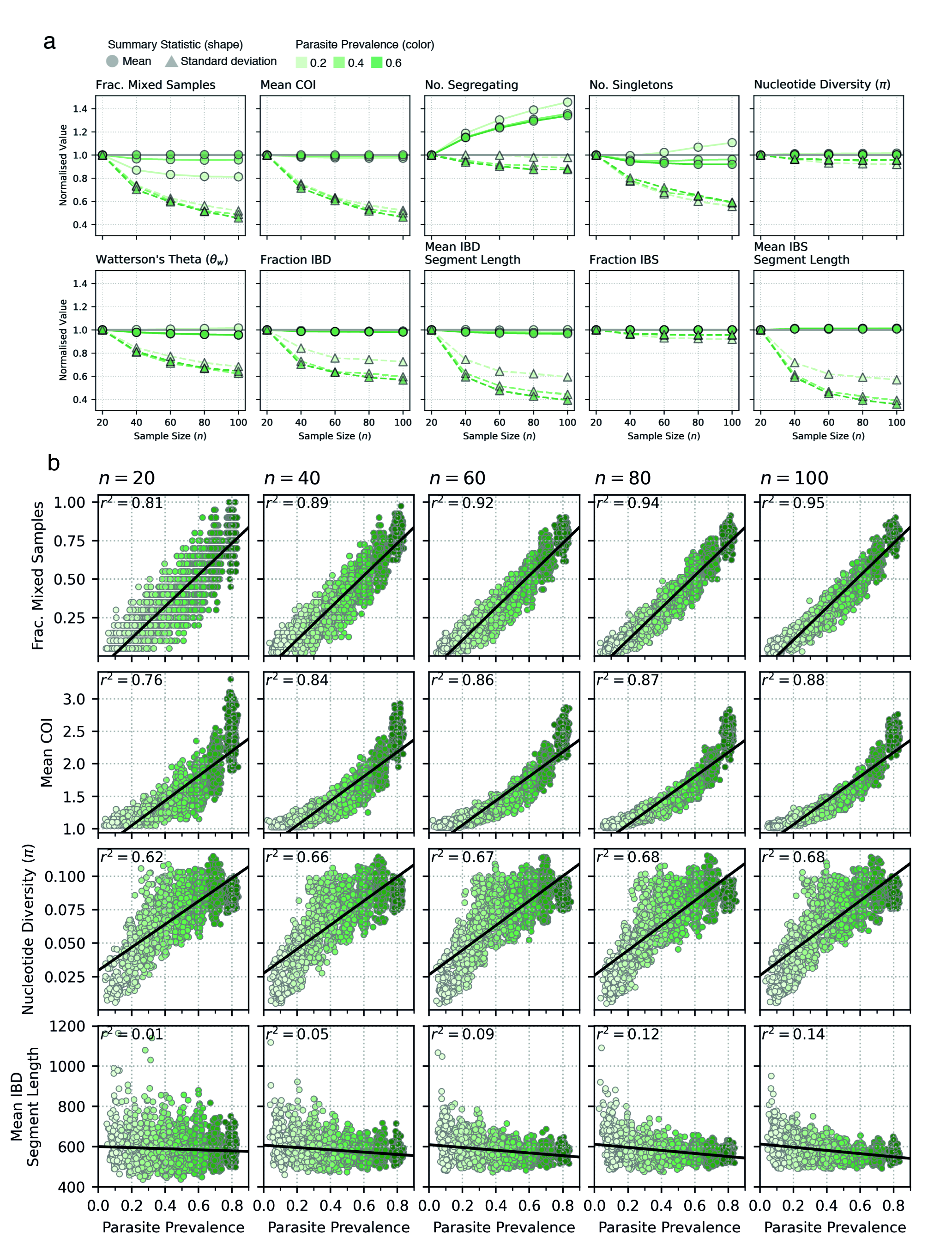

Supplement: S8 Fig — (a) Shows the influence of sample size on the mean and standard deviation of different genetic diversity statistics. The mean and standard deviation of each genetic diversity statistics across all samples collected (over ten years from thirty replicate simulations) is shown, for three different prevalence levels (indicated by color). Prevalence was varied by changing the number of vectors (Nv), and the mean and standard deviation are normalised to their value at a sample size of twenty. Note how increasing sample size reduces the standard deviation of different statistics to varying degrees. (b) Change in r2 for increasing sample sizes. Each row is a different genetic diversity statistic and each column a different sample size (indicated at top by n). Individual points within scatter plots represent estimates of the given genetic diversity statistic from samples of the indicated size, after simulations have reached equilibrium. Shades of green indicate the equilibrium prevalence values of individual simulations. r2 values for all genetic diversity statistics can be found in Fig 3b. (TIF) [file pcbi.1009287.s009.tif]

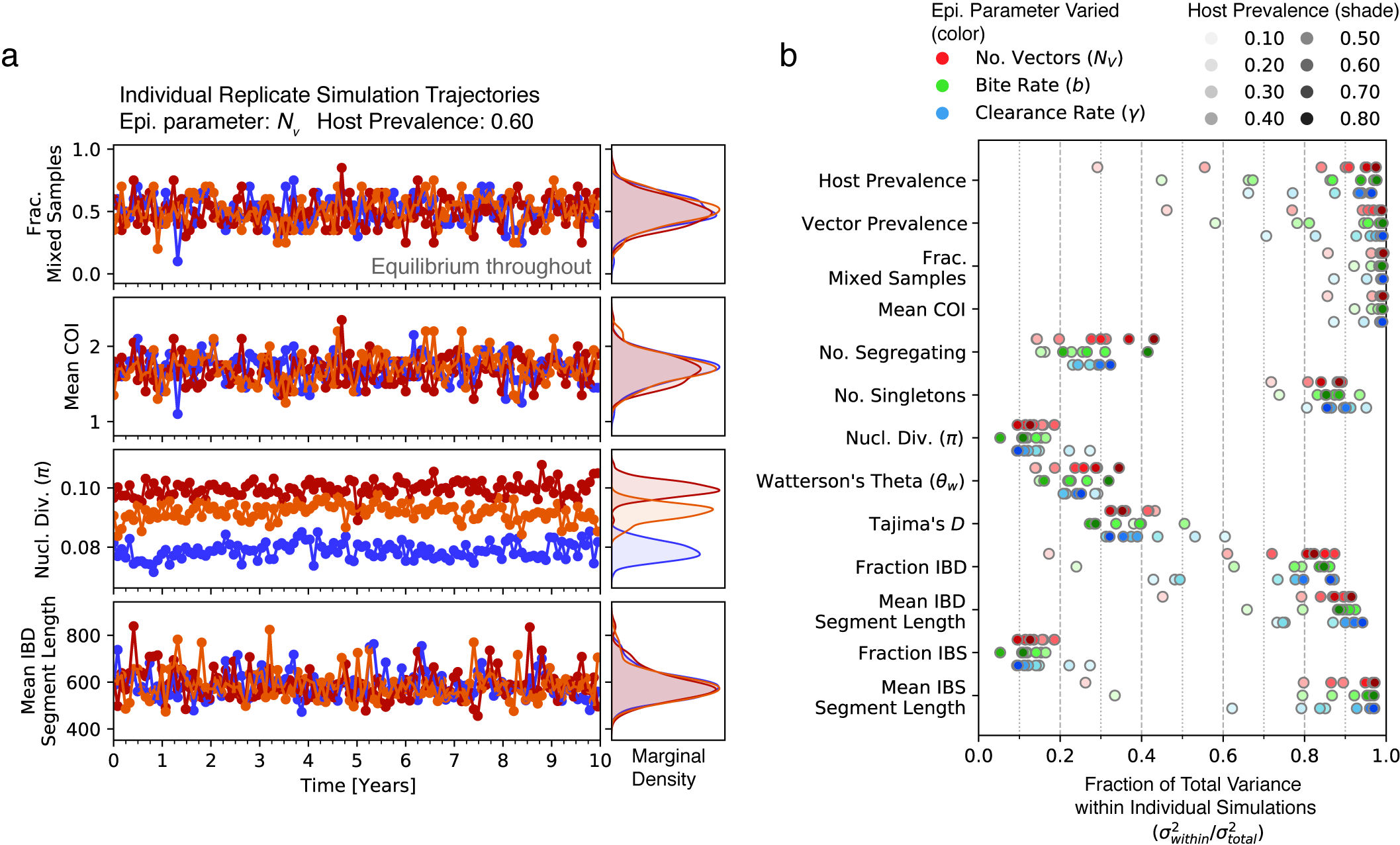

Supplement: S9 Fig — (a) Trajectories of a set of four genetic diversity statistics over a ten year period at equilibrium, for three randomly selected replicate simulations (indicated by color). Each replicate simulation has the same host prevalence of 60%, achieved by tuning the number of vectors Nv. Marginal densities for the diversity statistics in each replicate simulation are shown at right. Note how for nucleotide diversity, there is substantial variation between replicate simulations; whereas for other statistics most variation is observed within individual simulations. (b) The fraction of the total variance occurring within- rather than between-individual replicate simulations is shown for all genetic diversity statistics. Each point represents a particular statistic (y-axis), varied epidemiological parameter (color) and equilibrium prevalence value (shade). Analysis is conducted over thirty replicate simulations for each epidemiological parameter and prevalence level. (TIF) [file pcbi.1009287.s010.tif]

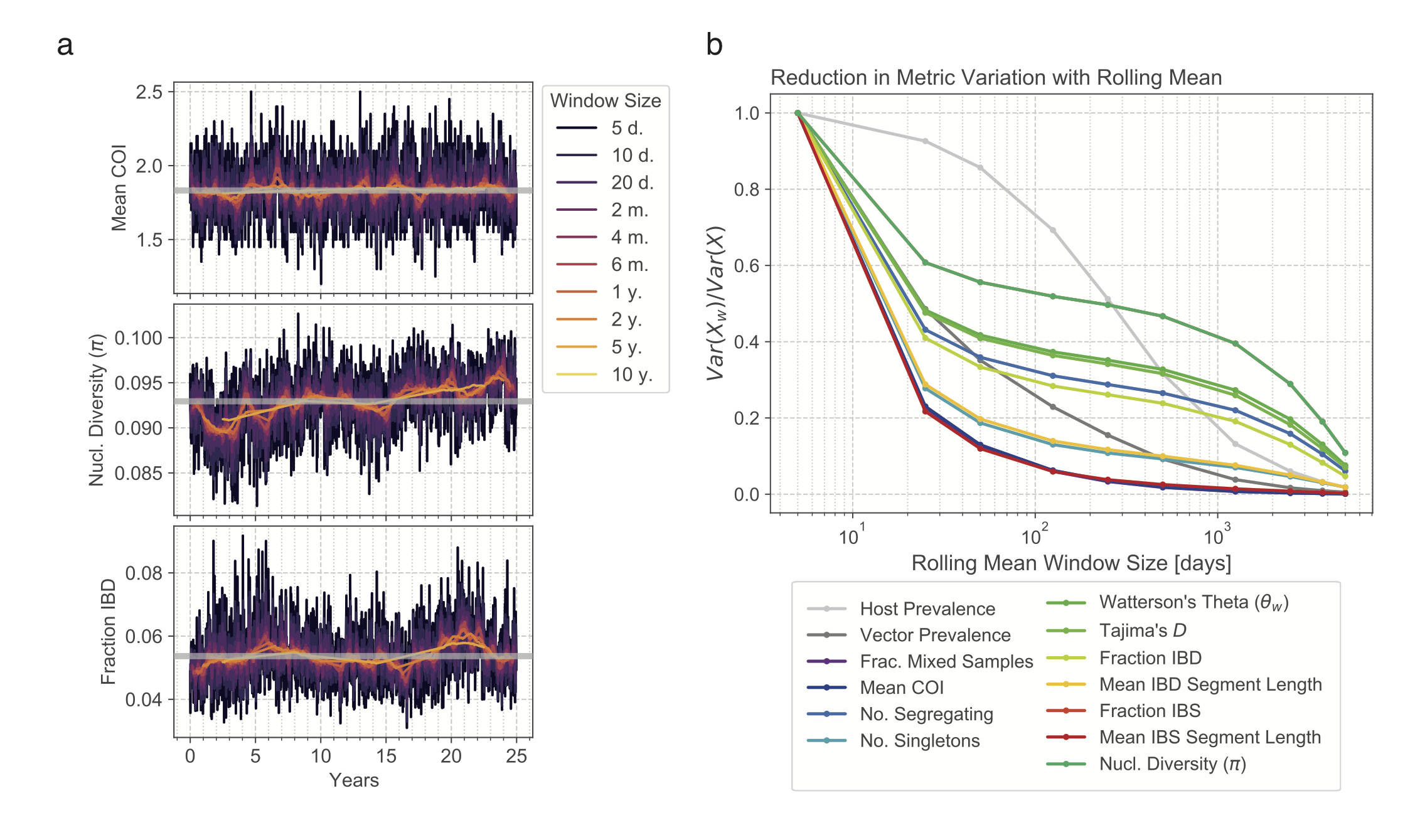

Supplement: S10 Fig — Panel (a) shows the noisy behaviour of three genetic diversity statistics (y-axis) from an individual simulation where parasite prevalence was kept fixed at 0.65 for a 25 year period (x-axis). The trajectory of each statistic was smoothed using a rolling mean, with window sizes varying from 1 day (1 d., purple), which is equivalent to no smoothing, up to 10 years (10 y., yellow). The mean of the statistic during the 25-year window is indicated with the grey horizontal bar. Notice how even with a 10-year window rolling mean, the nucleotide divesity still deviates from its mean value. (b) Across 100 independent replicate simulations, the reduction in variance of each genetic diversity statistic with increasing rolling mean window sizes is shown. The y-axis gives the ratio of the variance for the window size indicated by the x-axis (Var(Xw)) divided by the unsmoothed variance in the genetic diversity statistic (Var(X)). Increasing with window size of the rolling mean always reduces the variance, but at different rates for different statistics. (TIF) [file pcbi.1009287.s011.tif]

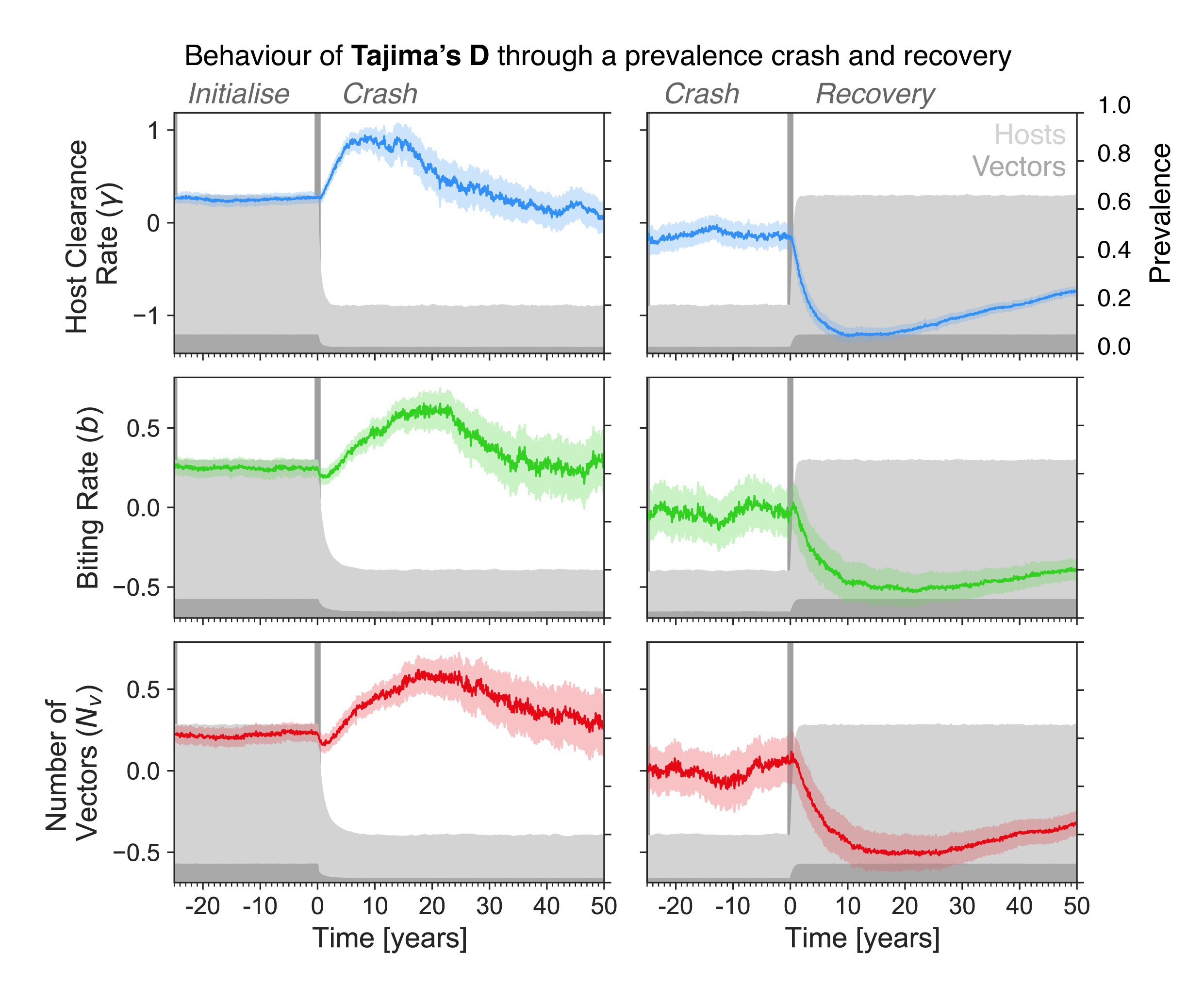

Supplement: S11 Fig — Colored lines show mean estimate across 100 replicate simulations, shaded area gives 95% confidence intervals. Notice how Tajima’s D increases during a population contraction and decreases during population growth. (TIF) [file pcbi.1009287.s012.tif]

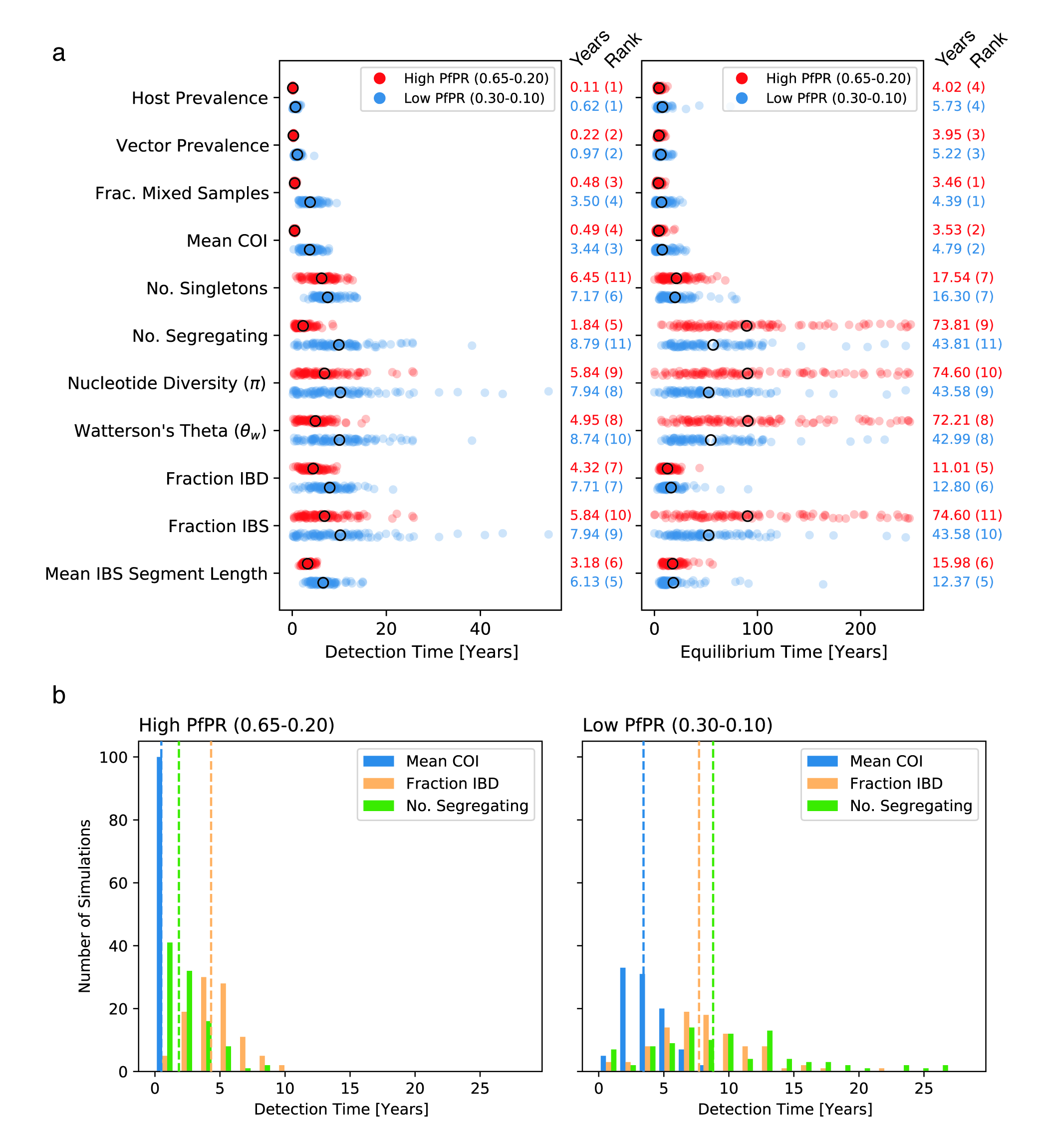

Supplement: S12 Fig — (a) Distributions of detection and equilibrium times are shown for experiments where the prevalence change was from 30% to 10% (Low PfPR, blue) or from 65% to 20% (High PfPR, red). Each point represents one of one hundred replicate simulations for each experiment. In both cases, prevalence was changed by reducing the number of vectors. Black circle indicates median. (b) Histograms of detection time distributions for Mean COI (blue), Fraction IBD (yellow) and Number of Segregating Sites (green). Vertical dashed lines indicate medians. Notice how the Fraction IBD has a faster median detection time when decline starts at 30%. (TIF) [file pcbi.1009287.s013.tif]

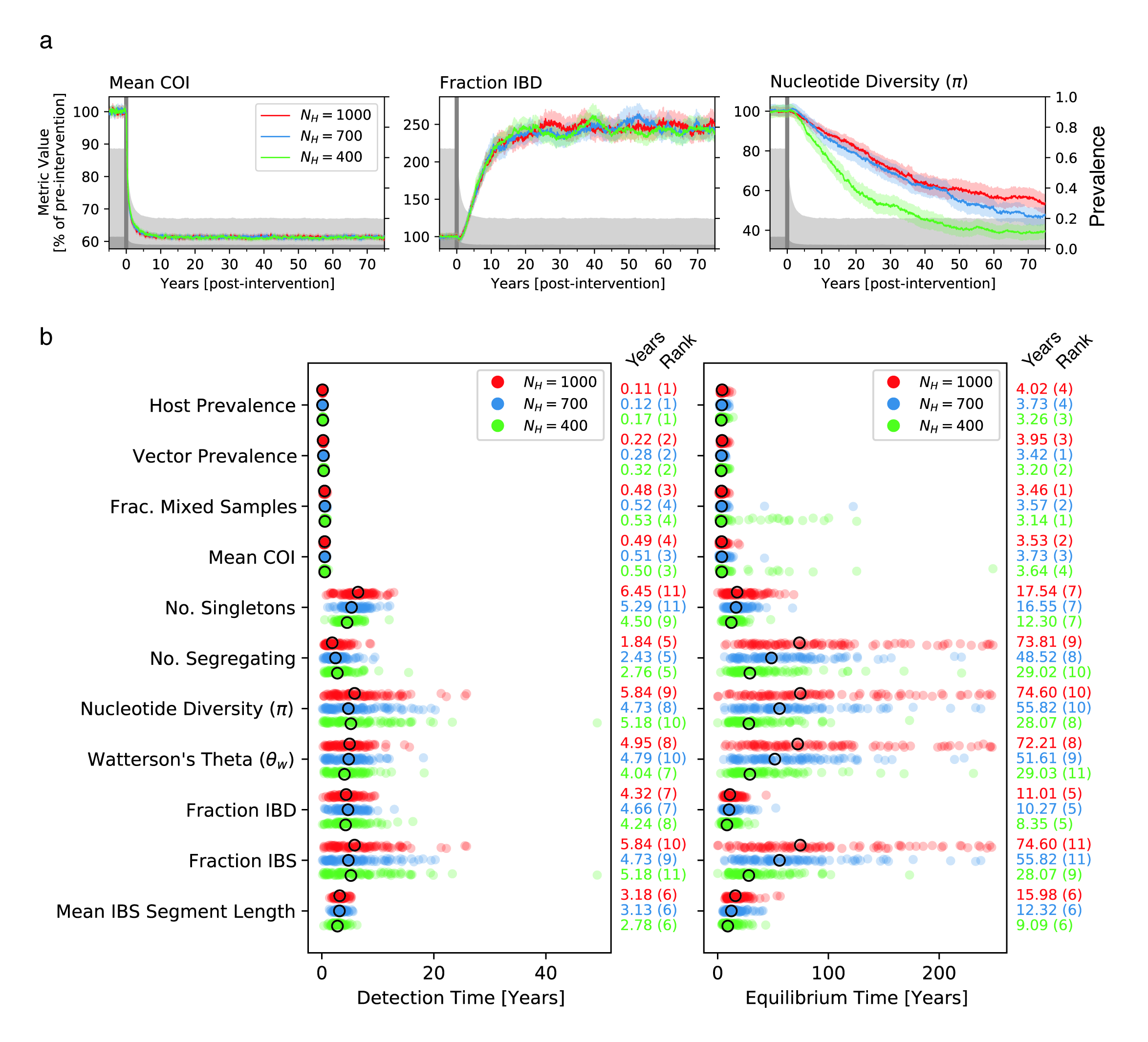

Supplement: S13 Fig — (a) The trajectories of Mean COI, Fraction IBD, and nucleotide diversity (π) are shown following a reduction in the number of vectors. Colored lines indicate average metric behaviour across 100 replicate simulations, with shaded area indicating standard error of the mean. Host population sizes of 1000 (red), 700 (blue) and 400 (green) are shown. Metrics are scaled to represent percentage of their pre-intervention mean. (b) Distributions of detection and equilibrium times for all statistics across 100 replicate simulations for each host population size. Median is indicated by black circle. Text at right gives median in years and parentheses give rank amongst all metrics for that host population size. (TIF) [file pcbi.1009287.s014.tif]

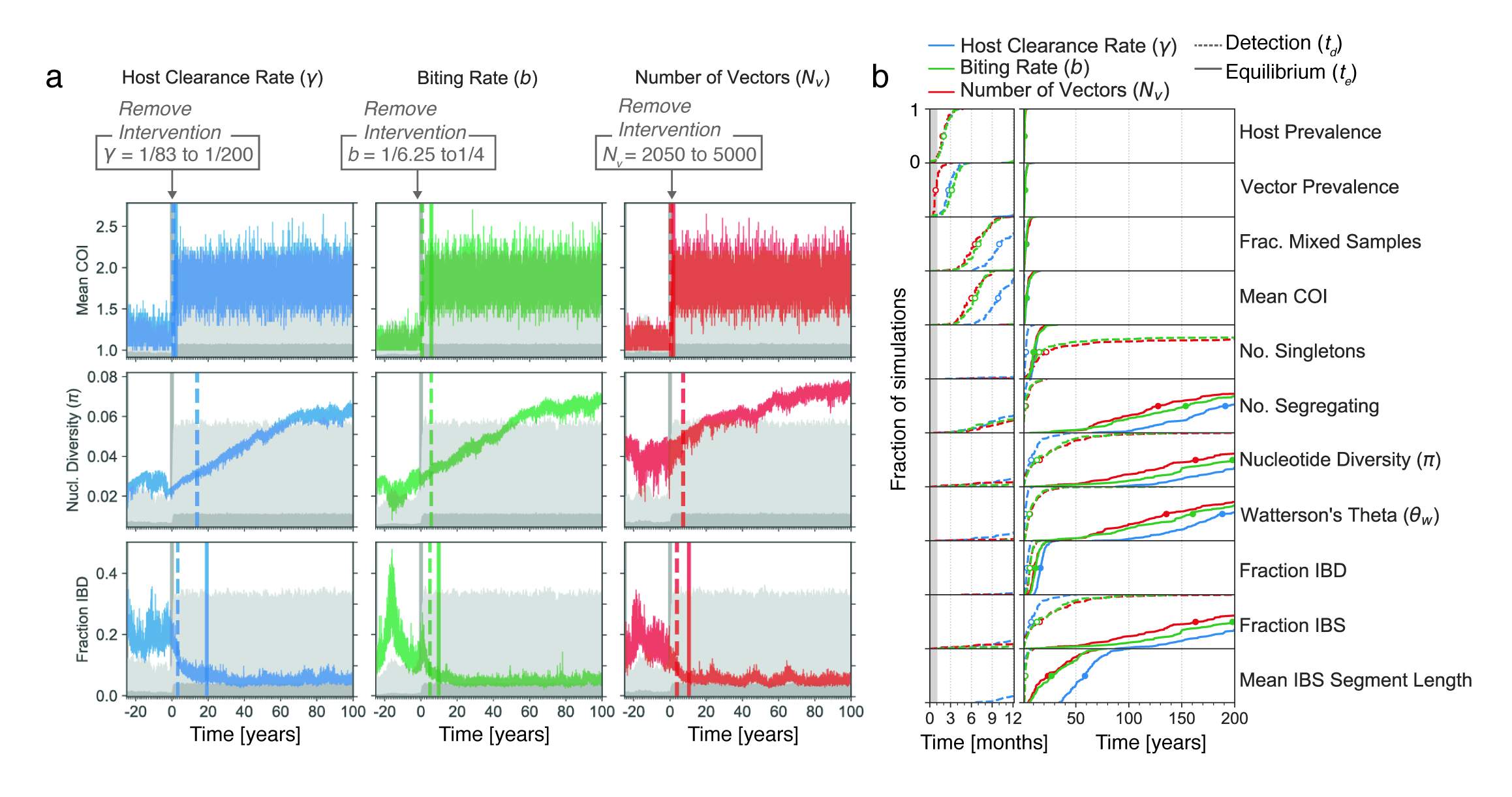

Supplement: S14 Fig — (a) Each plot shows the behaviour of a genetic diversity statistic in an individual simulation through a recovery of parasite prevalence, induced by: left column, reducing the host clearance rate (γ); middle column, increasing the vector biting rate (b); or right column, increasing the number of vectors. The intervention occurs at time zero (x-axis, grey vertical bar) in all cases. For each plot, the detection time (vertical dashed bar) and equilibrium time (vertical solid bar) of the genetic diversity statistic is indicated. Note that here a single simulation is shown for each intervention type. (b) Empirical cumulative density functions (ECDFs) of the detection and equilibrium times of diversity statistics, created from 100 independent replicate simulations for each intervention type. The y-axis gives the fraction of replicate simulations with a detection (dashed line) or equilibrium (solid line) less than the time indicated on the x-axis. Line color specifies the type of intervention. Open and closed circles give medians for the detection and equilibrium times, respectively. The first year is magnified for clarity. (TIF) [file pcbi.1009287.s015.tif]

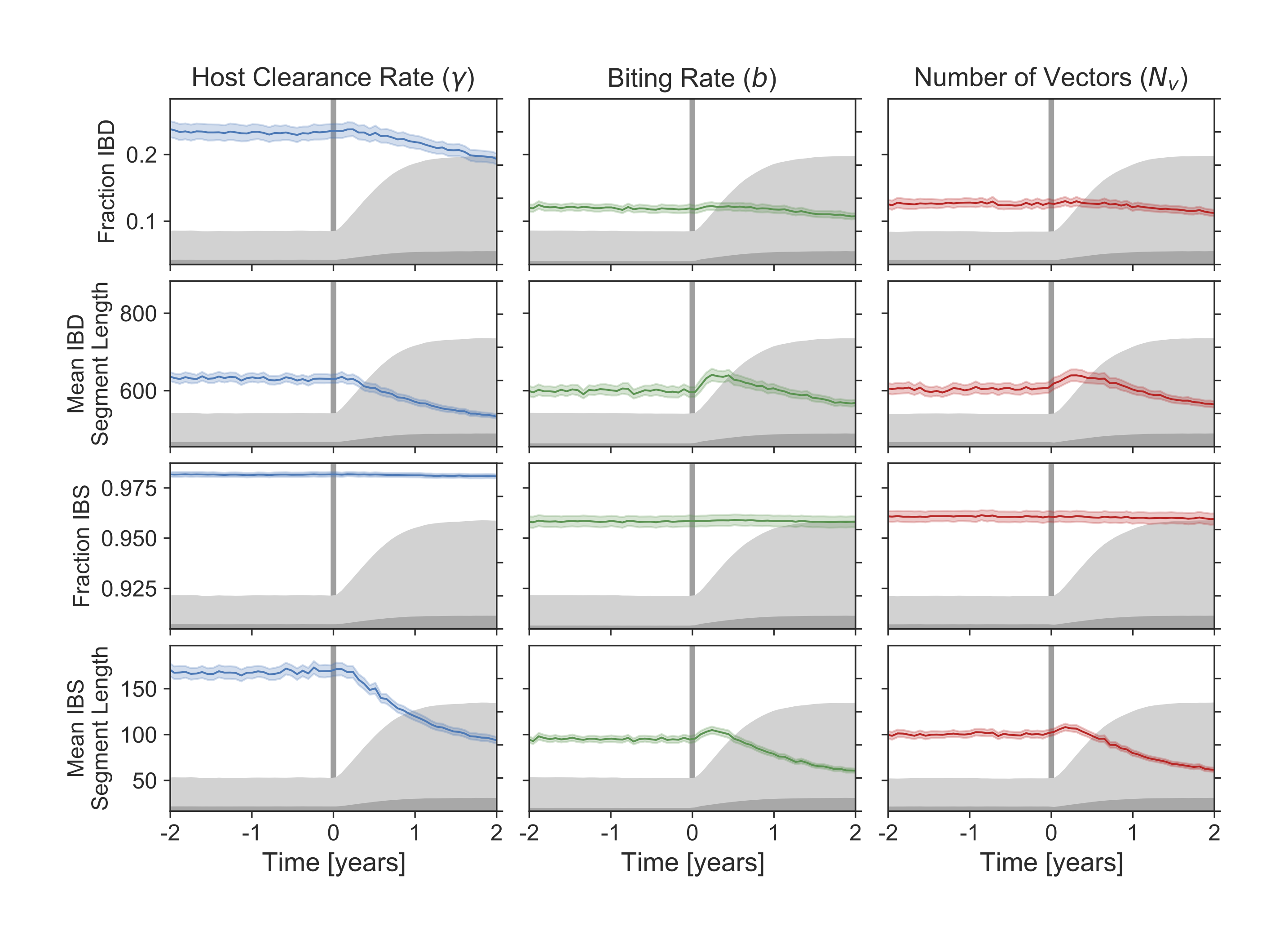

Supplement: S15 Fig — Focus on IBD and IBS statistics during a four-year window around the beginning of the recovery. Notice how for a change in the number of vectors (Nv) there is an increase in average IBS and IBD segment track length at the beginning of the recovery, consistent with epidemic expansion. (TIF) [file pcbi.1009287.s016.tif]

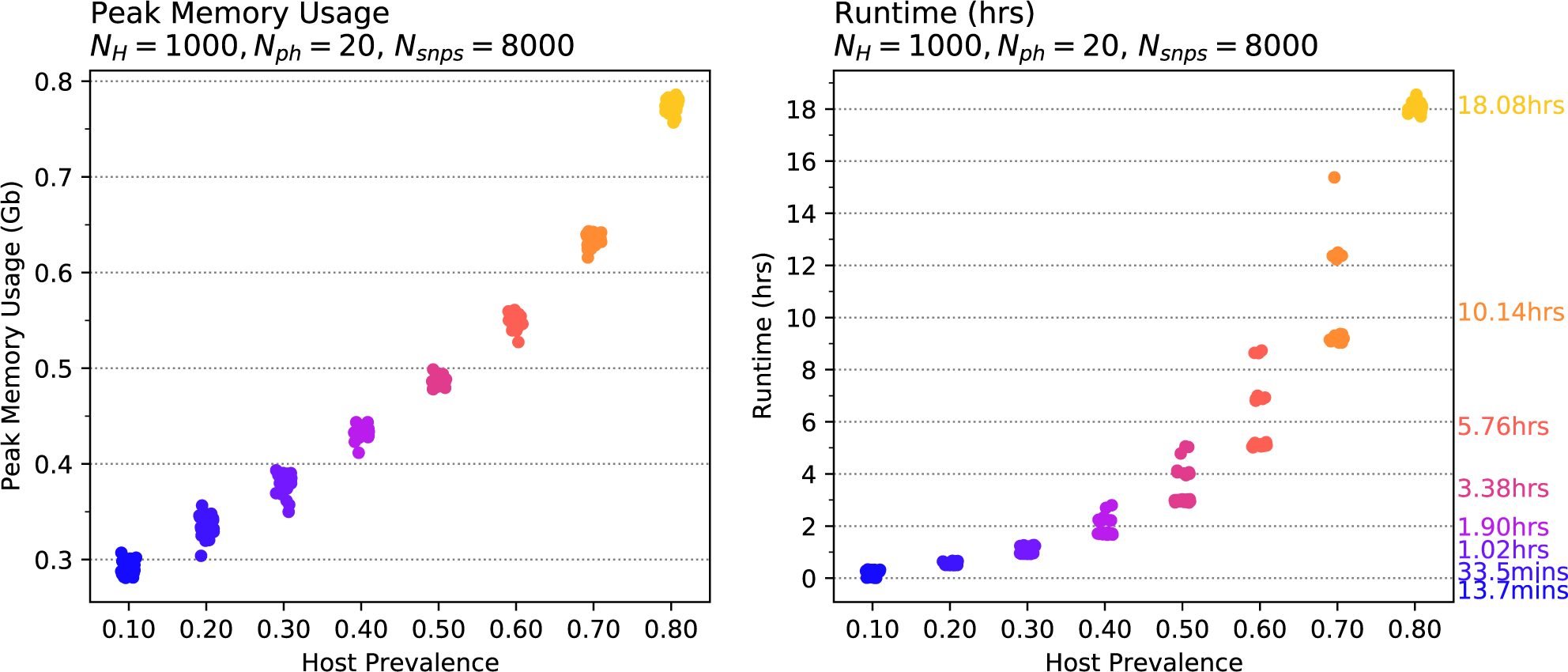

Supplement: S16 Fig — Left panel shows peak memory usage of individual forward-dream simulations brought to equilibrium at different prevalence values. For each prevalence value thirty replicate simulations are plotted; the same simulations analysed in 3. Right panel, same as left but showing run-times of individual simulations (run on 2.6GHz Intel Ivybridge CPUs with 15Gb RAM). Mean run times for each prevalence value are shown at right. (TIF) [file pcbi.1009287.s017.tif]
