# Supplementary material for: Elucidating relationships between P.falciparum prevalence and measures of genetic diversity with a combined genetic-epidemiological model of malaria
Source: PLoS Comput Biol. 2021 Aug 19;17(8):e1009287. doi: 10.1371/journal.pcbi.1009287 (PMC8407561; doi:10.1371/journal.pcbi.1009287)
Supplement: S1 Table — (PDF) [file pcbi.1009287.s018.pdf]

Statistic	Definition
Fraction Mixed Samples	Fraction of samples collected that contain > 1 distinct parasites
Mean Complexity of Infection (COI)	Mean number of distinct parasites per sample
Number of segregating sites (S_n)	Total number of polymorphic sites across all samples
Number of singletons	Number of polymorphic sites where the minor allele is present in only one sample
Nucleotide diversity (π)	Mean number of pairwise differences between two samples, normalized to the total number of sites
Watterson's Theta (θ_w)	Point estimate of $\theta = 2N_e\mu$ where μ is the mutation rate, given as $\theta_w = S_n / \sum_{i=1}^{n-1} \frac{1}{i}$
Tajima's D	A test statistic for the neutral mutation hypothesis Tajima1989
IBS Fraction	Mean fraction of sites identical between two parasites within the sample
Mean IBS Segment Length	Mean length of an IBS segment (≥ 1 consecutive identical sites) between two parasites within the sample
IBD Fraction	Mean fraction of the genome in IBD between two parasites within the sample. See Materials and Methods for details.
Mean IBD Segment Length	Mean length of an IBD segment between two parasites within the sample

S1 Table: Genetic diversity statistics computed in `forward-dream`.
