# Supplementary material for: Elucidating relationships between P.falciparum prevalence and measures of genetic diversity with a combined genetic-epidemiological model of malaria
Source: PLoS Comput Biol. 2021 Aug 19;17(8):e1009287. doi: 10.1371/journal.pcbi.1009287 (PMC8407561; doi:10.1371/journal.pcbi.1009287)
Supplement: Attachment — Submitted filename: r2r_fwd-dream.docx [file pcbi.1009287.s019.docx]

We thank the reviewers for their insightful comments. In response we have made major changes to both the simulation and manuscript. First, we greatly improved the computational performance of forward-dream to address several comments requesting simulations at lower prevalence values, with larger populations, and across a broader domain of parameter space (see image below). Second, we addressed comments directed at our super-infection model and the underestimation of COI by doubling the number of sub-compartments. Third, to address comments about the use of IBS instead of IBD, we now additionally estimate IBD using a length-threshold approach, which we have compared to an HMM implementation. Fourth, we have run additional permutations of the malaria control intervention experiments, from a lower prevalence and with a different population sizes, as suggested. Fifth, we have explored sources of noise at equilibrium, both in relation to sample size and stochastic features of the genetic genealogy. We acknowledge that other experiments were suggested, but we note that the total CPU time for the 1640 different simulations already conducted exceeds 29,600hrs (3.4yrs).

All of these changes are described in more detail below, as well as various textual changes to the manuscript and additional supplementary figures.

Figure 1. Speed improvements to forward-dream. Runtime in hours is compared between the original simulation (orange) and the simulation after updated in response to this review (blue). Each point represents an individual simulation run for 640 years (the time required to equilibrate at the highest prevalence value) with 400 hosts, 2000 vectors, and 10 within-host compartments; the simulation settings presented in the original manuscript.

**Reviewer #1**

I really enjoyed reading this paper and think it is a welcome addition to the slowly growing number of malaria transmission models looking at understanding parasite genetics. The study is well set out and explores commonly used metrics to explore the dynamics and utility of different metrics. I do have a few major comments I would like to see addressed and then some smaller minor comments.

Thank you for your review. We found it very helpful and have made improvements to both the simulation and manuscript as a consequence.

Major Comments

COI vs Prevalence Relationship

The COI prevalence relationship seems too low (i.e. not high enough COI at higher prevalences). Looking at estimates derived from THE REAL McCOIL (RMCL) we see evidence in Uganda of mean COI equal to ~7, ~5, and ~2.5. These are in three study sites in Uganda in the original THE REAL McCOIL study (see last table in their SI). RMCL has also been used to estimate COI in 5 countries in [https://www.nature.com/articles/s41467-020-15779-8 [nature.com]](https://urldefense.proofpoint.com/v2/url?u=https-3A__www.nature.com_articles_s41467-2D020-2D15779-2D8&d=DwMGaQ&c=D7ByGjS34AllFgecYw0iC6Zq7qlm8uclZFI0SqQnqBo&r=IOR1cDbzB3SbE2JhepXc6JXyFX9jEB9zvwQtQiFFuYc&m=DFqvrkwxTLsVMZXHsCP7lynonMY5RlSrQXadUG85ABU&s=6A-yL4ma2SoQEAU5708RDN64gHMM7FeXI-brozI_QUk&e=) and the mean COI estimated (see Supp Figure 3) also shows mean COI at least above 2. Also there is good literature on MOI by msp2 by prevalence (Fig 3 in[https://journals.plos.org/plosone/article?id=10.1371/journal.pone.0164054 [journals.plos.org]](https://urldefense.proofpoint.com/v2/url?u=https-3A__journals.plos.org_plosone_article-3Fid-3D10.1371_journal.pone.0164054&d=DwMGaQ&c=D7ByGjS34AllFgecYw0iC6Zq7qlm8uclZFI0SqQnqBo&r=IOR1cDbzB3SbE2JhepXc6JXyFX9jEB9zvwQtQiFFuYc&m=DFqvrkwxTLsVMZXHsCP7lynonMY5RlSrQXadUG85ABU&s=sjN7LWGEsu_wv8TsQ-iT8XtKFmX5V96FhnCASAv4L7E&e=)), which has been updated in[https://academic.oup.com/mbe/advance-article/doi/10.1093/molbev/msaa225/5902837 [academic.oup.com]](https://urldefense.proofpoint.com/v2/url?u=https-3A__academic.oup.com_mbe_advance-2Darticle_doi_10.1093_molbev_msaa225_5902837&d=DwMGaQ&c=D7ByGjS34AllFgecYw0iC6Zq7qlm8uclZFI0SqQnqBo&r=IOR1cDbzB3SbE2JhepXc6JXyFX9jEB9zvwQtQiFFuYc&m=DFqvrkwxTLsVMZXHsCP7lynonMY5RlSrQXadUG85ABU&s=mlhtbAA3FX-3V9F-p9QakuLGVPWVgKI1wu28rcPnoiw&e=). However, I think it would be easy enough to parameterise your model to this relationship.

We agree that the relationship between COI and prevalence that we presented in the manuscript was an underestimate relative to empirical observation.

We also appreciate that, at present, a majority of the COI estimates available in the literature are derived from RMCL, with values that tend to be significantly higher. At the same time, we have reservations about estimates from RMCL being considered ground-truth. First, we note that the model underlying RMCL has little power to resolve COI when it is high (see <https://malariajournal.biomedcentral.com/articles/10.1186/1475-2875-14-4> Figure 1b, d); that samples with COI > 4 will produce substantially overlapping probability mass distributions for the number of heterozygous sites, which is the signal leveraged by RMCL to make an inference. Second, RMCL infers COI using a Metropolis-Hasting algorithm with symmetric COI updates (+/- 1) and a uniform prior on COI in the range [0, 25]. In almost every case the prior on COI should be either monotonically decreasing or Poisson, and we expect that at a minimum, few researchers would agree that COI=25 is *a priori* as likely as a COI=1. These two features of RMCL can easily lead to scenarios where the *maximum a posteriori* COI is an overestimate. Consistent with this, the credible intervals RMCL reports around higher COI samples are very wide (see S2 Table).

Alternatively, methods we have previously developed to infer COI using whole-genome sequencing data (namely DEploid and DEploidIBD) have tended to produce much lower estimates of COI; though we acknowledge they also truncate the COI distribution – categorising samples with COI > 4 as COI=4.

To address this discrepancy, we have repeated the simulations but increasing the number of sub-compartments within hosts and vectors from ten to twenty, which has the effect of increasing average COI (though admittedly still not to the same levels as observed in RMCL analyses). We chose to hold the drift rate constant (though it could be reduced if one wanted an even higher COI relationship). The consequences of this are discussed in more detail in our response to your comment below ‘Superinfection Sub Compartments’.

IBD

Not sure how you are able to work out IBD if you are only tracking loci as 0 and 1. As you note, you are measuring identity by state. I would recommend not calling it IBD as it is incorrect and should be called IBS.

If you want to track IBD though it should be easy enough. Two approaches. Rather than encoding your genomes as 0/1 encode your loci with integers. (you may need larger than int8 for 400 individuals). Run your simulation until equilibrium prevalence and then replace each individual’s strains with integers, i.e. the first infected individual, all loci are encoded as 1, the next infected individual all loci are 2 etc all the way up to n. Then you are able to track ancestry and IBD relative to this time point. If you also store the genetic population at the time of replacing all your loci in this way, you can then use this as a look up table to work out the actual alleles. Then for mutations, when a mutation occurs, assign that compartment as the next integer value (n+1) and store this in your genetic population table (as well as that integer n+1 is a mutant integer and has the same identity as whichever integer the compartment was before the mutation event). This should then allow you to work out true IBD.

Alternatively, define a population level allele frequency for your 1000 loci. Then draw from those frequencies when seeding your population. Then you could estimate IBD using something like hmmIBD or deploidIBD and report that.

As it is currently, however, I do not agree with it being referred to as IBD and without seeing the comparison between IBD as above and IBS, I do not agree that we know trends in IBD will track with trends in IBS.

We have spent some time considering how to calculate IBD in our simulation instead of IBS. We considered your suggestions but identified some potential issues. The first approach -- where we assign an allelic identity to each genome at a given point in time -- would be hard to incorporate with longitudinal data sampling since, as the simulation moves forward in time, the ‘time after which alleles are considered distinct’ would move farther into the past and thus the IBD in the population would increase. We considered interfacing with hmmIBD, however the I/O and data engineering required would be cumbersome. DEploidIBD would be too slow and would also require simulating read data.

We explored two alternative approaches. The first was to implement a simple HMM of IBD within the simulation framework. The emission probabilities leverage information present in allele frequencies, similar to that implemented by hmmIBD. The second was to define IBD regions as being those with IBS in contiguous segments longer than 2cM, an approach similar to that of GERMLINE (https://www.ncbi.nlm.nih.gov/pmc/articles/PMC2652213/). We conducted small-scale experiments and found the two methods performed comparably for estimating mean IBD fraction across a large range of prevalence values (0.1 to 0.8). For estimates of IBD length, the methods tended to agree at lower prevalence values, but as IBD segments grow smaller at higher prevalence values, the length-threshold method plateaus at a longer mean segment length (likely as a consequence of genuine short fragments below the 2cM detection threshold). Despite this, we proceeded with the length threshold method as the computational costs of running a Viterbi on all pairwise comparisons between samples each time genetic data is collected were prohibitively high (several hundred times slower, even with a C-compiled version); and critically the length threshold method performs similarly in contexts were IBD is most relevant (i.e. low prevalence regions). You can see the full analysis in the GitHub repository at “notebooks/secR_revisiting-IBD-calculations.ipynb”.

Lastly, it is a shame that prevalence less than 20% was not explored as this is where I would expect measures of actual IBD to be most interest (For example, figure 4 in the isorelate methodological paper [https://journals.plos.org/plosgenetics/article?id=10.1371/journal.pgen.1007279 [journals.plos.org]](https://urldefense.proofpoint.com/v2/url?u=https-3A__journals.plos.org_plosgenetics_article-3Fid-3D10.1371_journal.pgen.1007279&d=DwMGaQ&c=D7ByGjS34AllFgecYw0iC6Zq7qlm8uclZFI0SqQnqBo&r=IOR1cDbzB3SbE2JhepXc6JXyFX9jEB9zvwQtQiFFuYc&m=DFqvrkwxTLsVMZXHsCP7lynonMY5RlSrQXadUG85ABU&s=KLQ2sxRUto4-pHBKQadd__TkfrQizIwe10IEKvgsUJg&e=) shows that it’s only really in SE Asia that you start observing higher IBD sharing). Alternatively, looking at within host IBD comparisons (Figure 4c in the deploidIBD paper [https://elifesciences.org/articles/40845#s2 [elifesciences.org]](https://urldefense.proofpoint.com/v2/url?u=https-3A__elifesciences.org_articles_40845-23s2&d=DwMGaQ&c=D7ByGjS34AllFgecYw0iC6Zq7qlm8uclZFI0SqQnqBo&r=IOR1cDbzB3SbE2JhepXc6JXyFX9jEB9zvwQtQiFFuYc&m=DFqvrkwxTLsVMZXHsCP7lynonMY5RlSrQXadUG85ABU&s=_huq-59bbZrYm3psx_KEdYeLJxutX5vIkV3eIm22WdI&e=)) shows the importance of these measures generally at lower transmission intensities. Perhaps worth thinking about if you do look at IBD.

With computational improvements to forward-dream we were able to simulate larger population sizes and thus explore prevalence values below 20% without regular stochastic extinction. To this end, we now include in the manuscript an equilibrium prevalence of 10% in Figure 3. However, since both runtime and memory now scale with total *infected* population size (the improved memory scaling is thanks to your suggestion), simulating much lower prevalence values is possible for those who are interested.

(Also I think it would be fine to say that it is not possible to look at IBD at this stage of fwd-dream, instead reporting IBS statistics and then discuss that this is something to be looked at in future versions).

Superinfection Sub Compartments

The use of sub-compartments to reflect parasites in hosts seems strange or I may have misunderstood something. Why does super infection have to replace previous parasites? I am not aware of any research suggesting that new infections cause old infections to be eliminated. Perhaps it might increase intra-host competition that would drive down the parasite density of older infections more quickly.

Related to sub-compartments. Why do you choose to always replace probabilistically half the genotypes in a superinfection? Should this not depend on the level of cotransmission and the genetic diversity of the incoming sporozoites? As well as perhaps the age/parasite density of present infections? For example, if the vector causing the infection was infected by an individual with a COI of 1, then only one genotype would be introduced but this one genotype being introduced could be causing a large reduction in COI to occur by replacing multiple genetic variants in an infected host that could have had previously a high COI. I wonder if this is one of the reasons for the low COI observed from the model as you are making the assumption that previous strains are lost upon a superinfection event.

Using a fixed number of sub-compartments -- and probabilistically replacing half of them upon super-infection -- were both modelling decisions that, we agree, are simplifications of reality. We discuss their motivations briefly below.

With regards to super-infection, we wanted the probability of fixation to be equal for both the pre-existing and incoming strains, i.e. we did not think there was any justification for assuming that the existing infection should have a competitive advantage over the strains being added to the pool by super-infection. In other words, conditional on the super-infection being established, we wanted to ensure that the old and new strains have an equal likelihood of persisting. In a neutral Moran model, the future fixation probability of an allele is equal to its present frequency. Thus, we initiate the super-infection by probabilistically replacing half of the existing strains.

However, you are correct that with this setup super-infection can in some cases lead to an initial reduction in diversity within the infection. Though intra-host competition could cause such an effect, we agree with you that there is no strong evidence for (nor probably neither against) such a mechanism. We have decided to minimise the likelihood that this occurs by increasing the number of sub-compartments within the infection to twenty. We still probabilistically replace half of all compartments, for the reasons outlined above. However, with twenty sub-compartments the probability of losing diversity is substantially decreased (e.g. for an allele with a 20% initial frequency, the probability drops from 34% to 12%). This can be easily shown analytically and we have verified the behaviour with the relevant simulation functions (see image below).

Figure 2. The probability of losing an allele during super-infection and its dependency on the number of sub-compartments. Here, we show the probability of losing an allele during a super-infection event (y-axis) as a function of the initial frequency of the allele within the host (x-axis) and the number of sub-compartments in the host (indicated by color). The fewer sub-compartments and the lower initial frequency, the greater the probability the allele is lost. The curves correspond to a binomial probability of drawing the allele zero times; here they have been created by simulation using the `infect_host()` function of forward-dream.

Timescales for metrics changing and crash dynamics

The discussion of timescales for metrics changing in response to changing prevalence is a really nice way of judging their utility. However, the reported time for metrics to change is somewhat dependent on the effective population size. You have quite a small population size and so I agree COI and other coinfection metrics will change quickly and relatedness metrics will change more slowly, the specific speed of this is really dependent on the effective population size. It would be good to redo this but with a larger and smaller population size (though may need to be 2 larger population sizes to make sure you can look at the same prevalence range).

We acted on your suggestion and repeated the crash dynamics with three population sizes (400, 700 and 1000 hosts). We found that the equilibrium times for many statistics increase with the size of the population (especially the *N_e_* dependent statistics). Interestingly, COI and Fraction IBD change only very slightly – another suggestion that they will be quite robust. Detection times also grow with population size but in a much less pronounced manner. We have a few sentences discussing this in the main results, and two supplementary figures (SFig 11 and 12).

The crash dynamics are quite strong, i.e. a large reduction in a short time span. This could be similar to maybe a period of an effective mass drug administration campaign. However, I would be interested to see the variance change in the metrics in response to a more gradual intervention campaign (similar to scale up of bed nets etc.) whereby we see prevalence change by say 30% absolute over 10 years? How useful are each metric in this more subtle change in transmission intensity and how reliably would these metrics be able to detect these changes?

We did an additional experiment where the decline starts from a lower prevalence value, but we have not conducted experiments with more gradual crash dynamics like those you describe. At present these experiments are quite expensive (~24hrs per simulation X 100s replicates). We think, with some feasible changes to the API, we could equilibrate the simulation a limited number of times and then conduct a variety of experiments, sweeping over different crash dynamics of interest with the same set of starting parasite populations. Unfortunately, we do not have the capacity to do this at present, though we agree the experiments would be interesting.

Other Comments

Throughout C.O.I. has been used as opposed to COI which is more common in the literature for complexity of infection. Would recommend replacing in order to align with the literature.

We have made this change.

In the introduction you mention the model developed for the Daniels et al Senegal study. I agree this is a bespoke model developed for a specific application and is not currently suitable in its current form for looking at other transmission dynamics. However, very recently a new transmission model for simulating malaria genetics was published - [https://academic.oup.com/mbe/advance-article/doi/10.1093/molbev/msaa225/5902837 [academic.oup.com]](https://urldefense.proofpoint.com/v2/url?u=https-3A__academic.oup.com_mbe_advance-2Darticle_doi_10.1093_molbev_msaa225_5902837&d=DwMGaQ&c=D7ByGjS34AllFgecYw0iC6Zq7qlm8uclZFI0SqQnqBo&r=IOR1cDbzB3SbE2JhepXc6JXyFX9jEB9zvwQtQiFFuYc&m=DFqvrkwxTLsVMZXHsCP7lynonMY5RlSrQXadUG85ABU&s=mlhtbAA3FX-3V9F-p9QakuLGVPWVgKI1wu28rcPnoiw&e=). This appears to address most of the requirements you note as necessary of an integrative genetic-epidemiological model. It would be good to reference this and also to then state the improvements/differences that forward-dream makes.

We now discuss both Daniels et al. and Watson et al. in our introduction. There are different ways forward-dream can be framed in relation to them. One perspective is that both these models exclude any within-host/vector evolution and have genetic models representative of small SNP barcodes, limiting the statistics they are able to calculate. The way the models are initiated (randomly) and equilibrated (for much less time than would be required for the most recent common ancestor to exist within the simulation) would also prevent the accurate calculation of many statistics, even if they included more SNPs. From this perspective forward-dream is quite a different type of model. A second perspective considers the spectrum of model complexity, with *Daniels et al.* and *Watson et al.* being at either end, and forward-dream somewhere in the middle.

Line 61: Replace ‘so-called “compartment-based”’ with simply ‘compartment-based’

We have made this change.

Drift model could also because of low COI. Perhaps this is also another way to parameterise the COI distribution by reducing the frequency of drift events. Also on the parameter estimate chosen for drift events, there may be some data you could use to estimate this perhaps. There are studies where infected individuals have had their parasites sequenced at various time points throughout an infection, often to test for parasite clearance vs reinfection events. I wonder if this information could be used to estimate observed COI in an individual over time, which could help identify drift event frequencies (in my head the time taken for drift events to remove a parasite lineage from a mixed infection could be the same as the time taken for a newly infected individual with a COI equal to 2 to be detected as a monoclonal).

Yes, we found the within-host drift parameter was one of the hardest to set; if it is influencing COI substantially one could change it without much hesitancy in principle. We also agree that the data you describe would be a good starting place for thinking about setting the parameter more empirically. However, for now we have adjusted the COI using the number of sub-compartments (which has the added benefit of preventing diversity loss during super-infection) and kept the drift rate as-is. We leave the challenge of making inference about within-host drift parameters to future work.

Fig3b where is the biting rate variation for tajimas and no. singletons?

Our apologies, here the results were obscured by over-plotting. We have added jitter.

I really enjoyed the discussion in the Supplementary on the “Probability a given parasite genome passes through the host-to-vector (ph) or vector-to-host bottleneck (pv)” about the methodological implications of bottlenecks at the vector-to-host bottleneck. I agree that with high sporozoite counts that it is likely that the majority of genetic diversity in the mosquito will be passed on. Might be worth though highlighting that if the SMFA results of ~5 sporozoites being passed on and defined by a geometric distribution is the correct distribution, then actually a 20% bottleneck will have substantial implications on diversity passed on as we would expect over 50% (cumulative density underneath geometric distribution with mean of 5 after removing zero counts as we are assuming infection is occurring) of infections to have between 1 and 5 sporozoites.

The number of sporozoites (~sub-compartments) that pass through the bottleneck is drawn from a binomial distribution – but your point still holds and is a good one. We have made mention of it in the supplementary material.

The parameterisation of prevalence is conducted using biting rate, vector abundance and clearance rate. I would think that vector abundance and clearance rate are the more realistic two rather than biting rate. However, these are only explored in parallel. When vector abundance increases and there is increased immunity in the population this then leads to an increase in clearance rate. Not sure it will change the results but would be interested to see how the dynamics in Figure 3b would look when both of these are changed at the same time.

We agree this would be interesting but we will leave it for future work.

As an aside, it would be interesting to see how forward-dream could be sped up and population size increased. Having gone through the code the first thing that springs to mind re memory is that you have your full human and vector model state as one 3 dimensional array, a lot of which will not be being used when individuals are susceptible. Would be great to see if this could be improved for future versions so that spatial dynamics etc could be explored between multiple populations etc.

In response to your and the other reviewers responses we have made a significant effort to increase the speed of forward-dream to facilitate larger population sizes. We took your suggestion (thank you!) and replaced the three-dimensional array with a dictionary of two dimensional arrays, and some rough memory profiling indicating that this reduced the maximum memory allocation by about 1/3 at a host prevalence of 66% (as expected). Clearly the savings will be much greater at lower prevalence values.

**Reviewer #2**

The manuscript is an attempt by the authors to fill an important gap in the malaria field – translating epidemiologic setting to expected genomic data by linking an epidemiologic and genomic model, the latter including within host as well as transmission elements. A useful model in the space would be quite welcome and have numerous immediate practical applications. The model set up and outputs are clearly described and the manuscript is overall well written, particularly given the potential layers of complexity. While all models are abstractions of reality, they need to at least **recover the main features of key relationships have any utility. Unfortunately, I am not convinced that the authors have surpassed this bar**, limiting my enthusiasm for what is otherwise a well-performed piece of work.

Thank you for your review. We are confident that we have captured the first-order effects, and constructed a useful model that sheds light on key relationships between metrics and prevalence, as we will discuss further below.

Major comments:

1) The stated goal of fwd-dream is to provide a basis for assessing the expected performance of methods for inferring transmission intensity from genetic data, and designing sampling strategies for different scenarios (paraphrased from lines 464-7 at the end of the discussion). To have any confidence in utility towards these goals, we would ideally see some model calibration and at a bare minimum at least some very basic “sanity checking” of model outputs with available empiric data on relationships between key transmission parameters and genetic outputs. Unless I have missed it, I do not see any attempt to do this in the manuscript and in fact some of the results presented appear to be red flags. In particular, the percentage of mixed infections and COI (both metrics which are widely, if not systematically, available in the literature) appear to be gross underestimates of what is empirically observed for a given level of parasite prevalence. This first becomes apparent in figure 2, where at 40% parasite prevalence we see only a ~5% prevalence of mixed infections which is much lower available empiric data I am familiar with. In figure 3 (and associated supplemental data) we see COI increase to a maximum of ~1.5 at 80% parasite prevalence, in which setting one might expect much higher COI in most of the population even just based on first principles i.e. nearly everyone is infected, most people are semi-immune and as such have long, untreated infections with considerable superinfection and coinfection. Because these relationships are not believable, it is difficult to know what to make of the other ones, or to be convinced of any of the conclusions regarding the different epidemiologic parameters having differential effects on genetic outputs. The result that metrics of within host diversity would change faster than population level metrics is probably true **but does not seem an open question to me given that infections last on the order of one year and most population genetic parameters would be expected to change at a much slower rate.**

First, we apologise because in response to this comment we have realised that Figure 2 may be misleading. The `Host Mixed` prevalence given in Figure 2 is the fraction of the *entire host population*, and not just the fraction of infected hosts – the fraction of infected hosts with mixed infections is thus 2.5X higher. A second point regarding Figure 2 is that it shows the initiation phase of the simulation, hundreds-to-thousands of years before equilibrium levels of diversity are established. As a result, the rate of mixed infection is somewhat lower, as super-/co-infection with very related strains can go undetected.

Despite these caveats, we agree that the relationship between prevalence and the rate of mixed infection was underestimated – this was also raised by another reviewer. We have addressed this by increasing the number of sub-compartments within hosts, and thus shifting the Mean COI and fraction of mixed infections upwards, above estimates produced by methods like DEploidIBD, but below those produced by THEREALMcCOIL.

Of course we also agree that calibration with data is ideal, however doing so correctly is a challenging problem that we feel can be presented separately from the new model we have built. It requires, first, both sufficient genetic data (in our case WGS) and associated metadata; ideally limited to a particular geography and collected longitudinal. Second, it requires a detailed understanding of local epidemiology and ecology in order to set model parameters – given the number of them in this type of model, overfitting is an issue that needs careful attention.

It is difficult to say if this is a fundamental flaw of the relatively simple model structure or a function of the specific parameters and limited population size/number of years for “burn-in” evaluated (which may derive from the amount of time needed to run the model). Regarding model structure, potential problems include not incorporating immunity, treatment, or age structure, heterogeneity, a limited and fixed number of within host compartments, and if I’m reading things correctly clearing all infections simultaneously as opposed to having independent clearance times for different parasites.

There is of course a spectrum of complexity along which models can be built; which end one favours is as much a matter of preference as principle. With forward-dream, being one of the first models in its class, we deliberately endeavoured to create as simple a structure as would capture the essence of malaria transmission and evolution. We incorporated mutation, intra-host, -vector and population-level drift, a malaria-specific model of meiosis, the transmission bottlenecks, super-infection and co-infection, and canonical vector-host infection dynamics. Moreover, we endeavoured to use, wherever possible, pre-existing “gold-standard” models – such as the Ross-McDonald and Moran – with a long-history of utility and a broadly-appreciated set of advantages and disadvantages.

Certainly, many or all of the phenomena you list could modulate the trends we have described. We don’t think incorporating all of them is necessary for utility, or imminently feasible. Even if we assume all of these behaviours can be reasonably recapitulated *in silico,* the resulting model would still require an enormous number of input parameter values. For example, the one other model we are aware of in this space is *Watson et al. 2020*, which lists 47 input parameters in its supplementary materials – and it is clear this is not even an exhaustive list, as it includes no parameters describing the actual genetic processes (i.e. mutation or recombination). If one believes that these features are all of critical importance, then one must also believe that correctly setting the parameters that condition their behaviour is of critical importance. Yet, who knows with confidence all these values? How to conduct sensitivity analyses on such a high dimensional space? Or how to fit them in the face of non-identifiability?

In the end, these complex models have as much or more assumptions than do simple ones – and require as much or more justification. We prefer simplicity, because even a non-technical reader will have the model structure, and consequently the assumptions, in hand – they can interpret and criticise the results accordingly. This is in contrast to complex models, where a full understanding of the structure and assumptions can elude even those with directly relevant experience. We believe that it is much harder to intelligently interpret their conclusions, and somewhat easier to be led astray.

Please note also that we elaborate on the simplifications of our model in the discussion. Finally, the supplementary materials includes a section exhaustively describing how each parameter value was set, with references to empirical data from the literature (where it exists) and an indication of our uncertainty where it is significant.

Re: run-time parameters, authors could consider a introducing a larger number of diverse parasites at the start instead of 10 identical parasites, allowing for more than 10 with

in host compartments (which seems like a good number but not if you are replacing half of them with every superinfection and trying to incorporate drift) a much larger host population size (400 seems quite low), running for hundreds or thousands of years instead of 25, etc.

As aforementioned we have increased the number of within-host and vector compartments to twenty. We also increased the host size in all experiments from 400 to 1000.

Starting with a large number of diverse parasite is unnecessary and potentially incorrect, as it could introduce bias into the equilibrium state of the parasite population. Rather, we started with a clonal population to ensure that all the genetic diversity we were analysing was created by the model itself – i.e. all of the mutations, the linkage structure, allele frequency spectrum, &c. was generated by the simulation – as opposed to being generated arbitrarily at initialisation. We did not run the population for 25 years to equilibrate (again our apologies as you may have been misled by Figure 2), we made an upper-bound estimate of the time required to reach equilibrium based on coalescent theory. This was done by estimating the time to most recent ancestor for the entire parasite population at epidemiological equilibrium, and ensuring the simulation was run for at least that long before we began our experiments. The specific time depends on the input parameters, but as a rule of thumb around two years of equilibration was allowed for every infected host in the population.

2) Another major limitation of the current model is that it only “works” at transmission levels consistent with 20% or greater parasite prevalence, and many of the metrics of interest to the community, e.g. those based on IBD, may only be expected to show relevant signal in areas of lower transmission.

We agree this was a major limitation of the model – it was due to computational constraints. We now have addressed these constrains, and can simulate arbitrarily low prevalence values – since the time-complexity scales as a function of the *infected* population size rather than the *total* population size. We have added simulations at 10% prevalence in the manuscript.

Minor comments:
3) The authors discuss a metric of IBD throughout the manuscript but I believe what they have actually calculated is IBS. This should be called IBS unless it is actually IBD being calculated.

We are now estimating IBD as well as IBS, using a length threshold approach. See response to Reviewer 1.

4) Some terms such as PR and EIR should be properly defined for clarity.

We now give definitions for R0, EIR, and PR in the introduction.

5) Some discussion of how the model could be fitted to real data and used to infer parameters, if this is the goal, should be included.

This is not the goal, however we have expanded our discussion of how forward-dream can be used with data.

6) The authors should consider justification of the simplicity of the transmission model, given the considerations above.

We have addressed this comment above.

**Reviewer #3**

Hendry et al. tackle a computational and theoretically difficult problem that has rarely been thoroughly addressed before. They aim to link a population-genetic model summarizing within-host genetic variation with a Ross-MacDonald epidemiology/ecological model. Notably, they use the model to study the impact of potential ecology and interventions on common genetic summary statistics. The goal of the model is not directly inference, but is instead an important contribution to our mechanistic and qualitative understanding of a complicated system.

Additionally, one of the most difficult and vague parts of related models is where the parameter values come from. The authors put together a thorough and easy to read summary with many citations and tables in the supplement, which itself is a useful contribution for other papers.

Thank you for your positive comments. We are happy to hear you believe the work in the supplementary materials will be useful to others.

Major comments

1. I appreciate model-based work as a means to understand general qualitative principles, but a main argument the authors make (especially in the introduction) is the need for inference and application to data. While data analysis is beyond the scope of the paper, it would be really beneficial to have a deeper discussion on the types of data, sampling schemes, inference methods, etc to make the model useful to empirically-driven investigators.

We have added an additional paragraph to the discussion covering how forward-dream could in future be applied to data and used for inference.

We were somewhat hesitant to include this originally, because we are not of the opinion that this is the best way forward (at least in the short term) for the simulation. Rather, we see the simulation as fundamentally an tool for exploration – one that can be used to motivate or justify other inference approaches, test hypotheses, calibrate intuitions, etc. Nonetheless we take your point that this will be a question in many readers minds and we have tried to address it.

2. how does the population size of 400, 100 replicate simulations, or sample of 20 individuals influence results? It seems like a computational decision rather than a biological one? The small sample size is particularly confusing given the high noise. While it is informative to try mimic sampling in data, it would be nice to compare the summary statistics calculated on the full 400 indvs to see where the stochasticity is coming from. Indeed, the authors close the discussion suggesting the tool be used when designing experiments—one of the biggest questions I could foresee the simulations helping with is choosing sample size to sequence under a limited budget, yet no attention is paid to sampling. For example, in Fig 3A, how much of the variation comes from the 30 replicate simulations and how much comes from subsamples of only 20 individuals?

We agree that this is a critical point and we have taken some steps to address it.

First, for our experiments at equilibrium, we have added a supplementary figure decomposing the variance for each genetic diversity statistic into between- or within- simulation fractions (SFig 8). You will see here that this depends largely on the statistic under consideration, with the variance in COI being mostly captured by individual simulations, whereas statistics like nucleotide diversity vary considerably between replicate simulations. Ultimately this variation reflects timescale over which these metrics fluctuate.

Second, we have expanded the equilibrium analysis to explore sample sizes of 20, 40, 60, 80 and 100 (Fig 3b and SFig 9), allowing us to understand the extent to which sampling contributed to the variation we observed in different statistics. Some statistics exhibit substantial (and analytically predictable) reductions in variance with larger samples sizes (for example the fraction of mixed samples, which is well modelled by a binomial proportion). For other statistics, such as nucleotide diversity, the majority of the noise we observe is unrelated to sampling. We cover various sources of noise and their relative contributions as an additional paragraph in the discussion.

3. Figure 3 shows the equilibrium behavior as a function of parameters that are known to vary or humans can directly influence. However, it is also important to understand how parameter choice for other parameters influences model outcomes and if for example error in one of those produce a signal of similar strength as intervention/biologically varying parameters. Said another way, how much does it matter if parameters such as recombination rate are inaccurate?

Again we agree this is a very good point – and one that we had thought about while drafting the manuscript. At that time, we considered performing something like a one-at-a-time (OAT) sensitivity analysis on the input parameters. In the end we felt that since the statements we are making in this manuscript mainly concern the relative behaviour of statistics (rather than absolute predictions) there would be little gained by what would be a very computationally expensive exercise; it would also further complicate the manuscript. We do take parameter values very seriously though, which we hope is reflected in the work that is documented in the supplementary materials.

Minor comments
•The approach used to study equilibrium behavior is interesting, namely matching the parasite prevalence rather than perturbations in the parameters. I agree this in some ways makes comparisons more equal and interpretable, but it seems important to know the parameter values (perhaps as supplementary information) that are required to produce such parasite prevalences. Are they reasonable?

All of the parameter values used to create the prevalence changes are displayed in SFig. 3. We believe they cover a reasonable range. For example, bounding the prevalence range of 0.1 to 0.8, we have the number of vectors ranging from ~1800 to ~9000; the mean biting rate from ~1/3 days to ~1/6 days; and the mean host duration of infection from 83 days to 350 days. Note that both the biting rate and host duration of infection are exponentially distributed so the majority of the density is below the mean.

•Brief summary of the recombination rather than citation of their previous paper would help.

We have elaborated on the model – see the last paragraph of the subsection ‘Infection Layer’

•A number of the intervention timings seem quite dependent on parameters chosen. This would be worth discussing

We have now expanded the section looking at intervention timings and consider different population sizes and initial starting prevalence values.

•DNA sequencing seems to be simulated in a way that only decreases diversity/increases IBD by not differentiating similar strains, but doesn’t account for processes in the other direction such as sequencing error. Is this because with the 5% cutoff similarity, most error would not produce a sequence called as a new strain? How much of an effect on summary statistics does the initial sequencing process make?

The reason we impose this threshold is because an infected host will frequently carry very near identical strains across many of its sub-compartments (i.e. a handful SNPs produced during that host’s infection, amongst thousands of positions). If no sequencing process was included, the number of strains collected from a host would equal the number of sub-compartments, which would produce unrealistic values for COI, IBD, nucleotide diversity, &c; such a process is necessary and the values of statistics beforehand are not very meaningful. This is also true to nature – a host will carry potentially trillions of parasites, but in typical infections most will only differ by one or two SNPs (ignoring regions like *var*). With a finite sequencing depth, this diversity will often be reduced to *K <=* 4 consensus sequences (e.g. with DEploidIBD).

With regards to sequencing error, our primary motivation for omitting it was simplicity. As you point out, with our threshold it would not produce new strains, however it would alter the SFS to some degree by increasing the apparent number of rare variants. In future work it would be easy enough to incorporate some sort of error model if a researcher had a specific sequencing platform / type of genetic data in mind.
