# Supplementary material for: Elucidating relationships between P.falciparum prevalence and measures of genetic diversity with a combined genetic-epidemiological model of malaria
Source: PLoS Comput Biol. 2021 Aug 19;17(8):e1009287. doi: 10.1371/journal.pcbi.1009287 (PMC8407561; doi:10.1371/journal.pcbi.1009287)
Supplement: Attachment — Submitted filename: r2r_r2_fwd-dream.docx [file pcbi.1009287.s020.docx]

We would like to thank Dr. Amy Goldberg and the two anonymous reviewers for their continued time and feedback. Both Dr. Goldberg and Reviewer #2 have no further major comments. Reviewer #1 has three major comments which we summarise below, and address in more detail in the response section.

Major comment #1 is a continuation from the first round of revisions, requesting us to further increase our relationship between COI and prevalence. Our response to this in the first round was to increase our COI relationship by between 40 - 60% at higher prevalence values, such that at a prevalence of 80% the median Mean COI is now as high as ~2.5 (when biting rate or the number of vectors is varied). This was below the estimated COI levels from some of the references provided by Reviewer #1, but consistent with other available empirical estimates of COI. To explain our decision, we raised the point that COI inference methods can have substantial uncertainty (manifest in the confidence intervals) associated with very high COI inferences, and that different methods disagree about the frequency of such high COI samples; yet of course, the estimated mean will be sensitive to them.

Major comment #2 and #3 are both new to this round of review. Major comment #2 requests we look at comparisons between prevalence and EIR, as a way to choose parameter sets for varying prevalence that will preserve the log-linear relationship observed in analyses of empirical data. Major comment #3 is that our Fraction IBD statistics are too high, and that we adjust how we run the model to bring them in line with existing IBD relationships that the reviewer suggests we should collect.

All three of these criticisms have a unifying theme – concern about the parameters under which our simulation model is run. Setting parameters correctly is an important and challenging problem, and we appreciate all critical thinking about how to assess whether parameters are well set. However, we already devote part of the discussion to this as a limitation, and furthermore we have a lengthy supplementary material describing all of the parameters in the model, how their values were selected, and where uncertainty exists, with references to data from the literature.

Moreover, the importance of highly accurate model calibration / parameter setting is a function of application. Here, we do not make any predictions about specific geographies, and we explicitly state in the discussion that the model in its current form is not suitable for direct inference.

Despite this, we still feel our manuscript will be of interest to readers of *PLoS Computational Biology*. In our view, it provides:

1. A new, unique, and publicly available model for an important research area where few models exist.
2. An assessment (largely qualitative) of the relative value of different genetic diversity statistics for malaria surveillance; at a time when using genetics for surveillance is a topic on the agenda of many research groups and NMCPs.

Our response to this round of revision has been to make several additions and clarifications in the discussion, to ensure the points raised by Reviewer #1 and our position with respect to them will be clear to all readers. Within the discussion, we stress the importance of exploring parameters so as to better match specifics of a particular location, though also note that this is not a replacement for formal inference.

Reviewer's Responses to Questions

**Comments to the Authors:
Please note here if the review is uploaded as an attachment.**

Reviewer #1: Thank you to the authors for their revised manuscript. It is much improved and encouraging to see the speed increase. I still have some major concerns though about how accurate the model is in terms of replicating malaria epidemiology and consequently some of the genetic relationships observed. I understand the model tries to be simple (no heterogeneity/immunity) and is still useful for detailing the mean behaviour. However, as a result it is important that the model reflects current evidence and the literature in terms of replication the observed relationships between malaria prevalence, EIR, COI and IBD.

The following are major comments that need to be adequately addressed.

1. The COI vs prevalence relationship has been improved but is still not sufficiently high. The author's response rebutting the validity of the RMCL at accurately estimating COI at high COIs is not to me fair. The authors claim it struggles greater than 4, but that is for COIL - the performance of RMCL is notably more capable at resolving COIs >4, but I will agree that it will struggle at accurately determining say COI > 8. The comments about the prior are not relevant - a flat prior is used because although we know COI is very unlikely to be as high as 25, we know that they can be high (because biting heterogeneity is very overdispersed) and so the flat prior is simplest and unlikely to be altering the inferred COI because it is by definition an uninformative prior. And although RMCL does not have the power to distinguish well between high COIs, it is not acceptable to use this as an argument to have parameterised the COI vs prevalence relationship to have the mean COI still only equal to 2 at 80% prevalence. For example, msp2 MOI literature I referenced in my initial review shows this. Also your comment about DEploidIBD showing lower COIs is because DEploidIBD fails with COIs above 4. For evidence of this, see the recent single cell analysis from Malawi here[https://www.sciencedirect.com/science/article/pii/S1931312819306304 [sciencedirect.com]](https://urldefense.proofpoint.com/v2/url?u=https-3A__www.sciencedirect.com_science_article_pii_S1931312819306304&d=DwMF-g&c=D7ByGjS34AllFgecYw0iC6Zq7qlm8uclZFI0SqQnqBo&r=IOR1cDbzB3SbE2JhepXc6JXyFX9jEB9zvwQtQiFFuYc&m=dF-NfNuHCsYbvvyrwYsrcfJxgWhY7RvaAN_515Lky2I&s=FED2NJiUrfcqE1-DSxX_QL_mkMZ_5dIy0BOq0GKP_4o&e=). In fact, RMCL run on these same samples actually gives higher COIs than DEploidIBD. The relationship between prevalence and COI should still be higher and should scale exponentially given the relationship between EIR and prevalence.

We note that there is a difference of opinion between ourselves and the reviewer on the relevance of the prior for RMCL. Moreover, we believe that there are several reasons as to why the revisions we have already carried out address this point.

First, the relationship between COI and prevalence reported in our simulations is broadly consistent with some – if not all – elements of the existing literature, including a metanalysis of *msp2* estimates (<https://journals.plos.org/plosone/article?id=10.1371/journal.pone.0164054>, Fig. 6b & Supplementary Table 1), the recent work with Molecular Inversion Probes (MIP) in the DRC by *Verity et al.* (<https://www.nature.com/articles/s41467-020-15779-8>, Supplementary Figure 3, 4b), and finally the COI estimates across thousands of globally collected WGS samples produced by MalariaGEN (<https://elifesciences.org/articles/40845>, Table 1 and Figure 7, plus unpublished data), with which we have considerable experience.

Second, there is considerable variability in empirical estimates of COI in the literature, even in cases where estimates are specifically expressed as a function of prevalence. This is, in part, a result of the limitations and biases which any inference approach possesses, whether it be based on length polymorphisms in microsatellites or antigenic surface genes, heterozygous sites in SNP barcodes, within-sample allele frequencies in WGS data, or consensus haplotypes in targeted amplicon sequencing. However, as shown here, the specific nature of the relationship will also depend on underlying epidemiological drivers – as well as the broader environmental context, levels of background genetic diversity, sampling strategy, etc.

Third, forward-dream is a simulation tool whose value is primarily in understanding qualitative features of relationships, rather than making precise quantitative inferences. The software is available on GitHub and its parameters have been carefully documented. We made a substantial effort to describe all of the parameters in the model in a lengthy section of the supplementary materials – not just their model definitions, but their interpretations, uncertainties, and what literature data was used to set them. They are further described in the GitHub repository, and are fully specified by a single configuration file (see <https://github.com/JasonAHendry/fwd-dream/tree/master/params>), which makes them simple to inspect and change. Therefore, if researchers have differing opinions about levels of COI or any other matter, we hope we have enabled them to modify parameter values easily and intelligently, and run simulations in whichever way they feel is most realistic.

In summary, we don’t feel that requesting us to once again increase the COI to prevalence relationship is justified given the variability in existing empirical data, the clear differing opinions about COI that have resulted from this, and the free availability of the simulation and parameter descriptions which enables others to use the tool how they see fit. It would require us to re-run all the simulations in the manuscript and re-generate all the figures, at a very substantial human- and compute-resource cost. Of course, in the future if we move the model towards applications that involve direct inference, we will certainly ensure to align parameters and relationships to the relevant context.

2. EIR outputs. Please could EIR vs prevalence relationships be shown. The relationship between EIR and prevalence is well studied and should be approximately log-linear ([https://malariajournal.biomedcentral.com/articles/10.1186/s12936-015-0864-3 [malariajournal.biomedcentral.com]](https://urldefense.proofpoint.com/v2/url?u=https-3A__malariajournal.biomedcentral.com_articles_10.1186_s12936-2D015-2D0864-2D3&d=DwMF-g&c=D7ByGjS34AllFgecYw0iC6Zq7qlm8uclZFI0SqQnqBo&r=IOR1cDbzB3SbE2JhepXc6JXyFX9jEB9zvwQtQiFFuYc&m=dF-NfNuHCsYbvvyrwYsrcfJxgWhY7RvaAN_515Lky2I&s=Wo4P-X0GZXsHbfQIuRn6B8t0voX6frQCvi3csUC1PWs&e=)). I don't think this will be true for a number of the simulations you have done in which prevalence is achieved by altering the clearance rate and keeping the biting rate low. I want to see this relationship as although you have shown that you can recreate different prevalence settings by changing one of three parameters, we have good evidence to know that the biting rate of mosquitoes is unlikely to be as low as 1 feed per 6 days and is closer to 2-3 days climate depending. I think if you constrain this parameter you should be able to identify a set (or range of sets) of the other two that give a suitable EIR vs prevalence relationship as well as a suitable COI vs prevalence relationship (if you don't want to believe the RMCL one then the msp2 literature).

We have plotted the EIR vs prevalence relationships and share this with you below, though we do not feel it is pertinent to the manuscript. The results are all reasonably straightforward mathematical consequences of the RM model: varying biting rate or number of vectors produces a log-linear trend between EIR and prevalence; varying host clearance rate produces a linear trend between EIR and prevalence (host clearance rate only influences EIR via the proportion of infected vectors, which is approximately linear with host prevalence). Interpreting this, just as the relationships between genetic diversity and prevalence depend on underlying epidemiological factors, so too will the relationship between EIR and prevalence. However, whereas illustrating the genetic dependency required building a genetic-epidemiological model, for EIR this is clear from the RM model alone – it is not particularly relevant to this manuscript.

The observation that aggregating empirical data across Africa results in a log-linear relationship between EIR and prevalence indicates only that geographical variation in prevalence is driven primarily by variation in vector density or biting rate. This does not mean that prevalence can’t vary due to other factors or processes. For example, the direct effect of mass drug administration would be to increase host clearance rate, not change vector density; longitudinal data collected over the period would likely deviate from a log-linear relationship. This is why in the paper we explicitly chose to vary prevalence in a variety of ways, to mimic various epidemiological possibilities of interest and explore their consequences. We make no statements about the relative frequency of these modes of prevalence variation in Africa or elsewhere.

To ensure that readers are clear on how to interpret these experiments, we have added to the discussion a section emphasising these points.

3. Could you plot the relationship between mean pairwise IBD fraction of samples within host against the proportion of mixed infections. I.e. what does it look like in comparison to Figure 4c in the DeploidIBD paper - [https://elifesciences.org/articles/40845 [elifesciences.org]](https://urldefense.proofpoint.com/v2/url?u=https-3A__elifesciences.org_articles_40845&d=DwMF-g&c=D7ByGjS34AllFgecYw0iC6Zq7qlm8uclZFI0SqQnqBo&r=IOR1cDbzB3SbE2JhepXc6JXyFX9jEB9zvwQtQiFFuYc&m=dF-NfNuHCsYbvvyrwYsrcfJxgWhY7RvaAN_515Lky2I&s=jdnNujRs035p5fly8psbADlteBi2h4mfOp8M55HR7is&e=). The population level pairwise IBD seems very high. At 20% prevalence the Fraction IBD seems close to 0.25, which would mean that on average each parasite is a half sibling of a randomly chosen parasite in the population. That cannot be correct. Have I misunderstood? Is Fraction IBD the fraction of the genomes in mixed infections that are IBD? If it is the population level mean IBD, then this needs to be again addressed to better match relationships observed for IBD. There are a number of studies that have used hmmIBD to estimate population level IBD which would be a starting point - collecting these and plotting the relationship between these and malaria prevalence and compare your relationship (which I thinks shows that fraction IBD is too high).

The Fraction IBD statistic presented in the manuscript is the mean pairwise fraction of the genome estimated to be in IBD across all pairwise comparisons for the sample of genomes collected, including within- and between- host comparisons (i.e. the mean of the pairwise matrix including all recovered genomes). How all genetic diversity statistics were calculated – including the IBD statistics -- is described in the Materials & Methods, and statistic definitions are provided in Supplementary Table 1. As a consequence, readers should be able to interpret them without ambiguity.

In terms of the absolute value of this statistic, the first factor to consider is that the simulated population is quite small -- 1000 total hosts – and is also completely closed. Thus, at 10 or 20% prevalence there are only 100 or 200 infected hosts, of which we are sampling 40. Moreover, the population will have always been this small; the diversity that we simulate is created by equilibrating a population of a fixed size; not by, for example, simulating migration from a larger and more diverse population (as might occur in nature). This is one reason why the absolute values of IBD at low prevalence are higher than one might expect.

The second factor to keep in mind is that IBD is always defined with respect to an arbitrary number of generations in the past, and this directly influences absolute values. In this case, we went with the standard of defining IBS segments longer than 2cM as IBD -- which corresponds to the expected length of a track diminished by fifty intervening generations of serial outbreeding and associated recombination. Importantly, observing that 25% of the genome meets this standard does not immediately imply that a pair of genomes are half-siblings -- it depends on the breeding history, and a critical consideration is the length of tracks across which the IBD is distributed. If you look at the length of the tracks at the lower prevalence values (10 and 20%), they are quite small, much smaller on average than the expectation for half-siblings. This is because they were likely picked up through inbreeding events that occurred in the intervening generations, as a consequence of the small population size.

We have added to the discussion to make this clear to all readers.

---

Thank you for the changes made so far and it is encouraging to see the speed gains that have been made so far. Also thank you for keeping your code and analysis notebooks public - has made it much easier to understand what is going on. I look forward to reviewing the manuscript again with these comments addressed.

Reviewer #2: The authors have gone to some length to improve the model performance, analysis, and interpretation. The manuscript is improved and I have no remaining major comments. It is still difficult to know to what degree the method will be useful in exploring any quantitative relationships as opposed to broad, qualitative ones, but the authors defer more quantitative evaluation / calibration for future work. The authors now state directly in the discussion section that the goal of the model at least at present is not e.g. for ABC type estimation but for broad stroke evaluation of relationships, and they have done a sufficient job to justify that use.

If the authors do embark on more quantitative analysis in the future I still feel somewhat comfortable in stating that available empiric data in the literature suggest that the relationship with COI is substantially underestimated with the current model/parameters, hindering potential inference. The authors state in response to Reviewer 1 that the majority of estimates have been using The Real McCOIL and suggest there may be issues with overestimation with this method, but this is a recent approach based on availability of SNP data and for many years the most common way of evaluating within-host diversity (and likely representing the majority of existing literature) has been through genotyping of a limited number of polymorphic markers (initially via length polymorphism of antigens and microsatellites and more recently via amplicon sequencing). These methods may still over or underestimate COI, but most available data including more recent amplicon sequencing data suggest much higher COI. A few references on quick search:

Recent data using amp seq, see supplemental fig 3a and 3b: [https://pubmed.ncbi.nlm.nih.gov/31819062/ [pubmed.ncbi.nlm.nih.gov]](https://urldefense.proofpoint.com/v2/url?u=https-3A__pubmed.ncbi.nlm.nih.gov_31819062_&d=DwMF-g&c=D7ByGjS34AllFgecYw0iC6Zq7qlm8uclZFI0SqQnqBo&r=IOR1cDbzB3SbE2JhepXc6JXyFX9jEB9zvwQtQiFFuYc&m=dF-NfNuHCsYbvvyrwYsrcfJxgWhY7RvaAN_515Lky2I&s=fr3mssJnxb3rPH7BmB7U7y-84vu8mSr3ogFGKLxlv8Q&e=)
Using microsats, mean 3.4: [https://pubmed.ncbi.nlm.nih.gov/32246127/ [pubmed.ncbi.nlm.nih.gov]](https://urldefense.proofpoint.com/v2/url?u=https-3A__pubmed.ncbi.nlm.nih.gov_32246127_&d=DwMF-g&c=D7ByGjS34AllFgecYw0iC6Zq7qlm8uclZFI0SqQnqBo&r=IOR1cDbzB3SbE2JhepXc6JXyFX9jEB9zvwQtQiFFuYc&m=dF-NfNuHCsYbvvyrwYsrcfJxgWhY7RvaAN_515Lky2I&s=0cBKkmBg-HixOk_uiP1uPeKx_UGaVvUOuAnGr36AlTs&e=)
Table 4 here has some refs: [https://pubmed.ncbi.nlm.nih.gov/33172455/ [pubmed.ncbi.nlm.nih.gov]](https://urldefense.proofpoint.com/v2/url?u=https-3A__pubmed.ncbi.nlm.nih.gov_33172455_&d=DwMF-g&c=D7ByGjS34AllFgecYw0iC6Zq7qlm8uclZFI0SqQnqBo&r=IOR1cDbzB3SbE2JhepXc6JXyFX9jEB9zvwQtQiFFuYc&m=dF-NfNuHCsYbvvyrwYsrcfJxgWhY7RvaAN_515Lky2I&s=FskUPDRGHf8wF1TJ_xGVL97gWmvICMhqCIKwUyn-BtE&e=)
Table S1 here has some refs: [https://pubmed.ncbi.nlm.nih.gov/27711149/ [pubmed.ncbi.nlm.nih.gov]](https://urldefense.proofpoint.com/v2/url?u=https-3A__pubmed.ncbi.nlm.nih.gov_27711149_&d=DwMF-g&c=D7ByGjS34AllFgecYw0iC6Zq7qlm8uclZFI0SqQnqBo&r=IOR1cDbzB3SbE2JhepXc6JXyFX9jEB9zvwQtQiFFuYc&m=dF-NfNuHCsYbvvyrwYsrcfJxgWhY7RvaAN_515Lky2I&s=vFBVMpNxfriNgEF7vQQQaQVAlwjnAuDa4IIRWNaKH-Q&e=)

Thanks again for your comments and also for providing some additional references to empirical data on COI. For more discussion on COI please see our response to the first major comment of Reviewer #1.

Reviewer #3: The authors have spent considerable time and thought putting together a reasonable and thorough response to reviewer requests. I agree with them that their model, while (as always) an imperfect representation of the real world, is a big improvement to the current available models. More detailed models are susceptible to many assumptions based on processes and parameters we have poor estimates for. They also demonstrate biologically-relevant information that can be learned from their model.

Thank you very much for your comments and encouragement.
